# Insecticidal and Enzyme Inhibition Activities of Leaf/Bark Extracts, Fractions, Seed Oil and Isolated Compounds from *Triadica sebifera* (L.) Small against *Aphis craccivora* Koch

**DOI:** 10.3390/molecules27061967

**Published:** 2022-03-18

**Authors:** Shudh Kirti Dolma, Prithvi Pal Singh, Sajjalavarahalli G. Eswara Reddy

**Affiliations:** 1 Entomology Laboratory, Agrotechnology Division, CSIR-Institute of Himalayan Bioresource Technology, Palampur 176061, India; skdolma@gmail.com; 2 Academy of Scientific and Innovative Research (AcSIR), Ghaziabad 201002, India; 3 Chemical Technology Division, CSIR-Institute of Himalayan Bioresource Technology, Palampur 176061, India; thakurprithvi028@gmail.com

**Keywords:** *Triadica sebifera*, *Aphis craccivora*, residual toxicity, synergistic, enzyme assay

## Abstract

Aphid, *Aphis craccivora* Koch (Hemiptera: Aphididae), is a major sap-sucking insect pest of leguminous crops and also transmits plant viruses, leading to economic yield loss. Indiscriminate and repeated use of insecticides for control of aphid leads to the development of resistance, and is harmful to the environment, non-target organisms, etc. Plant-based extracts/seed oils (SO) are the best alternatives to insecticides. Insecticidal activities of *Triadica sebifera* have not been reported against *A. craccivora* and other insect pests to date. In the current study, the main objective was to study the insecticidal activities of leaf/bark extracts/fractions, seed oil, isolated compounds, and their combinations against *A. craccivora*. Results showed that, among the extracts, ethanolic bark extract 80% (LC_50_ = 5115.98 mg/L) was more effective against *A. craccivora*. Among fractions, the *n*-hexane fraction of leaves (LC_50_ = 425.73 mg/L) and the ethyl acetate fraction of bark (LC_50_ = 813.45 mg/L) were promising. Among compounds, gallic acid was the most effective (LC_50_ = 1303.68 mg/L) compared to shikimic acid and quercetin. SO (LC_50_ = 850.94 mg/L) was superior compared to extracts/fractions/compounds. All the combinations showed toxicity and synergistic activity. Leaf/bark extracts and SO significantly inhibited the AChE and GST activity in *A. craccivora*. Based on field bio-efficacy, the leaf extract/SO or their combinations can be recommended for the control of aphids.

## 1. Introduction

Aphid, *Aphis craccivora* Koch (Hemiptera: Aphididae), is the major sap-sucking insect pest of leguminous crops. Adults and nymphs also suck the plant sap from leaves/stem/pods, which affects the growth. In severe infestation, which leads to stunted growth of plants, secretion of honeydew on leaves/pods attracts the development of sooty mold fungus, which affects the photosynthesis of plants. *A. craccivora* also transmits plant viruses, leading to economic yield loss [1]. Currently, farmers are using synthetic insecticides of different classes for the control of aphids. Non-judicious and repeated application of insecticides for control of aphids leads to the development of resistance [2]. Due to the harmful effect of chemical insecticides on the environment, the public, the customers, and non-target organisms, there is a need of an essential substitute for the effective management/control of aphids.

Chinese tallow, *Triadica sebifera* (L.) Small (Euphorbiaceae), is the world’s most invasive species, common in China [3]. It is used for traditional medicine in China, Taiwan, and Japan. Due to the rapid growth and widespread invasion of native ecosystems, it is a popular ornamental species due to its beautiful foliage that changes from yellow to crimson color. Seeds of *T. sebifera* are used for herbal medicines, candles, soap, cocoa butter, lamps, heating oils, cosmetics, and pharmaceuticals [4,5]. The seed oil (SO) is used to treat skin disorders, purgatory, and root bark decoctions caused by dyspepsia [6]. The leaves and roots have depurative, diuretic, and laxative properties, and the decoction is used to cure edema and constipation. The root and bark extracts are used for the treatment of snake bites and skin ulcers [7], while the solvent leaf extracts/fractions/compounds have reported anti-bacterial [8,9,10], antimicrobial [9], and antioxidant properties [9,11,12].

Insecticidal activities of *T. sebifera* have not been reported against aphids and other insect pests to date. Therefore, in the present study, *T. sebifera* was explored for: (a) chemical composition of crude fat/oil and *n*-hexane fractions of leaf/bark ethanolic aqueous extracts, (b) isolation of compounds from ethyl acetate and *n*-butanol fractions of leaves, (c) residual toxicity of leaf/bark ethanolic/methanolic aqueous extracts, SO and its combinations, fractions, and isolated compounds, (d) combinations of SO with leaf/bark extracts for their synergistic interaction against *A. craccivora*, and (e) detoxification enzyme inhibition of ethanolic leaf/bark extracts and SO.

## 2. Results

### 2.1. Fatty Acid Composition of SO of T. sebifera 

The fatty acid composition and distribution of *T. sebifera* are presented in Table 1. The SO of *T. sebifera* affords a yield of 50% (*v*/*w*). The saturated fatty acids present in the SO were palmitic acid (4.99%) and stearic acid (1.38%.). The unsaturated fatty acids were oleic acid (8.78%), linoleic acid (15.42%), and palmitoleic acid (2.13%). The distribution of saturated fatty acids was less (6.73%) as compared to unsaturated fatty acids (26.33%).

### 2.2. Structure Elucidation of Isolated Compounds

Compound **1** was isolated as a white amorphous powder. The ESI-QTOF-MS of the compound showing the molecular ion peak at *m*/*z* 471.08 (M+Na)^+^ indicated the molecular formula as C_21_H_20_O_11_ (Supplementary Figure S1). Aromatic signals were observed in the proton spectrum at δ_H_ 7.98 d (2H, *J* = 8.4 Hz), 6.81 d (2H, *J* = 8.4 Hz), 6.30 s (1H), and 6.11 s (1H), showing the presence of two sets of equivalent protons and two singlet protons, which confirms the presence of two aromatic rings in the structure. Additionally, the presence of a doublet signal at δ_H_ 5.16 d (1H, *J* = 7.2 Hz), showing the HSQC correlation with δc 104.2, indicates the presence of a *β*-d-glucose moiety, which was also confirmed by COSY and HMBC correlations. The carbon value at δc 179.4 showed the presence of the C=O group. Hence, from the above spectral data and the comparison with literature data [18], compound **1** was identified as kaempferol-3-o-glucoside (Supplementary Figure S2).

**Kaempferol-3-o-glucoside:** White amorphous powder; 128 mg; mp. 178 °C; ESI-QTOF-MS (Positive) *m*/*z*: 471.08 (M+Na)^+^^1^H-NMR (600 MHz, CD_3_OD) (Supplementary Figure S3 and Table S1), ^13^C-NMR (150 MHz, CD_3_OD) (Supplementary Figure S4 and Table S1).

Compound **2** was isolated as a white amorphous powder. The molecular formula C_21_H_20_O_11_ was calculated after ESI-QTOF-MS, showing the molecular ion peak at *m*/*z* 487.08 (M+Na^+^) (Supplementary Figure S5). Compound **2** was identified by comparing observed data with literature-reported data [19]. After the comparison of observed data with literature data, compound **2** was identified as quercetin-3-o-glucoside (Supplementary Figure S2).

**Quercetin-3-o-glucoside:** White amorphous powder; 150 mg; mp. 226 °C; ESI-QTOF-MS (Positive) *m*/*z*: 487.08 (M+Na^+^). ^1^H-NMR (600 MHz, CD_3_OD) (Supplementary Figure S6 and Table S1), ^13^C-NMR (150 MHz, CD_3_OD) (Supplementary Figure S7 and Table S1). 

Compound **3** was obtained as a white powder. The molecular ion peak was observed at *m*/*z* 341.03 (2M+H)^+^ in ESI-QTOF-MS (Supplementary Figure S8), which indicates the formation of a non-covalent dimer in ESI-MS [20]. Hence, the stable molecular ion peak was formed in the form of a non-covalent dimer (2M+H)^+^. From spectral analysis and literature reports [21], compound **3** was identified as gallic acid (Supplementary Figure S2).

**Gallic acid:** White powder; 124 mg; mp. 260 °C; ESI-QTOF-MS (Positive) *m*/*z*: 341.03 (2M+H)^+^; ^1^H-NMR (600 MHz, CD_3_OD) (Supplementary Figure S9 and Table S2), ^13^C-NMR (150 MHz, CD_3_OD) (Supplementary Figure S10 and Table S2).

Similarly, compound **4** was also isolated as a white powder. The molecular ion peak for compound **4** was obtained at *m*/*z* 349.32 (2M+H)^+^ (Supplementary Figure S11), which was also the molecular ion peak of the non-covalent dimer peak of compound **4**. ESI-QTOF-MS analyses of compound **4** indicated its molecular formula as C_7_H_10_O_5_, and after analysis and comparison of observed data with literature data [22], compound **4** was identified as shikimic acid (Supplementary Figure S2).

**Shikimic acid:** White powder; 108 mg; mp. 184–185 °C, ESI-QTOF-MS (Positive) *m*/*z*: 349.32 (2M+H)^+^; ^1^H-NMR (600 MHz, CD_3_OD) (Supplementary Figure S12 and Table S2), ^13^C-NMR (150 MHz, CD_3_OD) (Supplementary Figure S13 and Table S2).

### 2.3. Gas Chromatography-Mass Spectrophotometry (GC-MS) Analysis of n-Hexane Fractions

The metabolites and their mass fragmentation, identified through GC-MS in the *n*-hexane fractions of leaves and bark, are presented in Table 2. In the *n*-hexane fraction of the leaves, the major metabolites were *n*-hexadecanoic acid (15.61%), followed by octadecanoic acid, ethyl ester (9.85%), and neophytadiene (5.87%). In the case of the *n*-hexane fraction of the bark, the major metabolites were galaxolide (44.73%), followed by ethyl phthalate (28.43%) and 1-octadecene (2.69%).

### 2.4. Residual Toxicity of Leaf/Bark Extracts and SO of T. sebifera against A. craccivora

The residual toxicity of LEE, LME, BEE, BME, and SO of *T. sebifera* against aphids with respect to LC_50_ values is presented in Table 3. Bark extracts were more effective than leaf extracts. Among leaf extracts, LEE 80% was found to be most effective (LC_50_ = 9590.49 mg/L) against *A. craccivora* after 72 h, followed by LME 100%, 80%, and 50% (LC_50_ = 9627, 10,800, and 11,540 mg/L, respectively), as compared to LEE 100% (LC_50_ = 14,100 mg/L). Similarly, 96 h after treatment, LEE 80% was found to be more effective (LC_50_ = 6756.42 mg/L), followed by LME 80% and 100% (LC_50_ = 7120.27 and 7528.56 mg/L, respectively), as compared to LME 50% and LEE 100% (LC_50_ = 7579.55 and 8702.07 mg/L, respectively).

Among bark extracts, BEE 80% (LC_50_ = 7300.57 mg/L) was most effective against aphids after 72 h, followed by BEE and BME 100% (LC_50_ = 8325.46 and 8737.64 mg/L, respectively), as compared to BME 80% and 50% and BEE 50% (LC_50_ = 9490.58, 10,580, and 10,650 mg/L, respectively). Similarly, 96 h after treatment, BEE 80% (LC_50_ = 5115.98 mg/L) was found to be more effective, followed by BEE 100% (LC_50_ = 5228.89 mg/L), as compared to BME 50%, 100%, and 80%, and BEE 50% (LC_50_ = 5233.81, 5701.69, 5779.72, and 7098.41 mg/L, respectively).

The SO of *T. sebifera* in the current study was found to be more effective after 72 and 96 h of treatment (LC_50_ = 2504.59 and 850.94 mg/L, respectively) than BEE and LEE (LC_50_ = 5115.98–6756.42 mg/L). All the leaf and bark extracts were not superior to the positive control, Indo-neem (Azadirachtin 0.15% EC) after 72 and 96 h (LC_50_ = 2642.32 and 1174.22 mg/L, respectively). However, the SO was superior to leaf and bark extracts.

### 2.5. Residual Toxicity of the Combination of SO with Leaf/Bark Extract of T. sebifera and Its Synergistic Activity against A. craccivora under Laboratory Conditions

The residual toxicity of the combination of SO with 80% LEE/BEE and its synergistic activity of *T. sebifera* against *A. craccivora* under laboratory conditions are presented in Supplementary Tables S3 and S4. Results showed that among the combinations studied, a mixture of LEE 80%+SO and BEE 80% {(1+1):2} was found to be more effective (LC_50_ = 239.94 mg/L) against nymphs of *A. craccivora* after 72 h, followed by mixtures of BEE+SO (1:1) (LC_50_ = 263.56 mg/L), SO+LEE at a 1:1 ratio (LC_50_ = 303.29 mg/L), and LEE+BEE+SO at a 1:1:1 ratio (LC_50_ = 337.33 mg/L), as compared to other mixtures/blends. Based on the fractional effect index (FEI), all the combinations/blends evaluated against *A. craccivora* showed synergistic interaction after 72 h. Among them, LEE+BEE at 1:3, 3:1, and 1:1 ratios showed the most significant synergistic interaction (FEI = 0.088, 0.092, and 0.122, respectively), as compared to other blends/combinations. Similarly, 96 h after treatment, BEE+SO (1:1 ratio) was found to be more effective (LC_50_ = 144.26 mg/L) against *A. craccivora*, followed by SO+LEE at a 1:1 ratio (LC_50_ = 168.9 mg/L), LEE+SO+BEE at a {(1+1):2} ratio (LC_50_ = 170.46 mg/L), and LEE+BEE at a 1:3 ratio (LC_50_ = 179.31 mg/L), as compared to other mixtures/blends. Based on FEI, all the combinations/blends evaluated against *A. craccivora* showed a synergistic effect. Among them, LEE+BEE at 1:3, 3:1, and 1:1 ratios showed significant synergistic interactions (FEI = 0.061, 0.070, and 0.100, respectively), as compared to other blends/mixtures.

### 2.6. Residual Toxicity of SO, Leaf/Bark Extracts and Their Binary Mixtures of T. sebifera and Their Synergistic Activity against A. craccivora under Plant Growth Chamber

Residual toxicity of SO, leaf/bark extracts, and their binary mixtures (1:1) of *T. sebifera* against *A. craccivora* under plant growth chamber conditions is presented in Supplementary Table S5. Results showed that the binary mixture of SO+LEE (1:1 ratio) was found to be more effective against *A. craccivora* (LC_50_ = 264.05 mg/L) after 72 h, followed by SO+BEE (LC_50_ = 362.76 mg/L), as compared to SO, LEE, and BEE (LC_50_ = 1100.22, 1840.84, and 5073.99 mg/L, respectively). Based on FEI values, both the blends (SO+LEE and SO+BEE) showed a synergistic interaction (FEI = 0.38 and 0.40) against *A. craccivora* (Supplementary Table S5). Similarly, 96 h after treatment, binary mixtures of SO+LEE were more effective against *A. craccivora* (LC_50_ = 223.82 mg/L), followed by SO+BEE (LC_50_ = 247.54 mg/L), as compared to LEE, SO, and BEE (LC_50_ = 685.47, 706.53, and 2328.79 mg/L, respectively). Based on FEI values, SO+BEE showed a synergistic interaction (FEI = 0.46). A binary mixture of SO+LEE showed an additive interaction after 96 h of treatment.

### 2.7. Residual Toxicity of Leaf and Bark Fractions of T. sebifera against A. craccivora

The residual toxicity of leaf/bark fractions of *T. sebifera* against *A. craccivora* is presented in Table 4. Results showed that, among leaf fractions, the *n*-hexane fraction (LC_50_ = 425.73 mg/L) was more effective than ethyl acetate and *n*-butanol (LC_50_ = 838.89 and 1527.84 mg/L, respectively), as compared to the water fraction (LC_50_ = 2702.82 mg/L) after 72 h. Similarly, 96 h after treatment, the *n*-hexane fraction (LC_50_ = 196.61 mg/L) showed more promising efficacy than ethyl acetate (LC_50_ = 367.75 mg/L), as compared to water and *n*-butanol fraction (LC_50_ = 864.68 and 1527.84 mg/L, respectively).

Among bark fractions, the *n*-hexane fraction (LC_50_ = 1659.98 mg/L) was more promising against *A. craccivora* after 72 h than water (LC_50_ = 3049.50 mg/L), as compared to *n*-butanol and ethyl acetate (LC_50_ = 3539.63 and 3629.52 mg/L, respectively). Similarly, 96 h after treatment, the ethyl acetate fraction was more effective (LC_50_ = 813.45 mg/L) than water (LC_50_ = 915.15 mg/L), as compared to *n*-butanol and *n*-hexane (LC_50_ = 1071.81 and 1130.95 mg/L, respectively). All the leaf and bark fractions were more superior than the positive control, Indo-neem (Azadirachtin 0.15% EC), after 72 and 96 h (LC_50_ = 2642.32 and 1174.22 mg/L, respectively).

### 2.8. Residual Toxicity of Isolated Compounds of T. sebifera against A. craccivora

The experimental results on the residual toxicity of the isolated compounds against *A. craccivora* with respect to LC_50_ values are shown in Table 5. Among the four compounds isolated from leaf fractions of *T. sebifera*, gallic acid was the most effective (LC_50_ = 1303.68 mg/L) against *A. craccivora*, followed by shikimic acid and quercetin-3-o-glucoside (LC_50_ = 1725.09–1855.93 mg/L), as compared to kaempferol-3-o-glucoside (LC_50_ = 3762.69 mg/L). With respect to percent of mortality, gallic acid at 5000 mg/L reported 98% mortality (F_4,49_ = 135.79; *p* < 0.0001), followed by shikimic acid, quercetin-3-o-glucoside, and kaempferol-3-o-glucoside (88%, 80%, and 58%, respectively) (F_4,49_ = 129.71, 106.32, and 61.00; *p* < 0.0001) (Supplementary Figure S14). All the tested compounds were not superior to the positive control, Indo-neem (Azadirachtin 0.15% EC), after 72 and 96 h (LC_50_ = 2642.32 and 1174.22 mg/L, respectively), except for gallic acid after 72 h (LC_50_ = 2339.69 mg/L).

### 2.9. Detoxification Enzyme Activities of LEE, BEE, and SO against A. craccivora 

**Figure 1:**
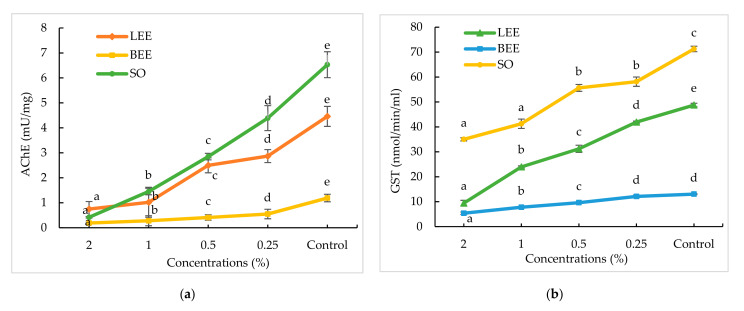
Detoxification enzyme activities: AChE (**a**) and GST inhibition (**b**) in *Aphis craccivora* treated with leaf ethanolic extract (LEE), bark ethanolic extract (BEE), and seed oil (SO) of *Triadica sebifera*. Bars represent standard error (±SE) of three replications. Means followed by the same letters within a column do not differ significantly by Tukey’s HSD test (*p* ≥ 0.05).

Detoxifying enzymes’ (GST and AChE) activities in *A. craccivora* fed with bean leaf discs treated with different concentrations of LEE, BEE, and SO are presented in Figure 1. Results showed that the AChE activity of nymphs of *A. craccivora* indicated that all the concentrations of LEE, BEE, and SO significantly inhibited the AChE activity (F_4,14_ = 631.00 to 968.50, *p* < 0.0001), as compared to the control. However, LEE, BEE, and SO at 2% reported higher inhibition of AChE (0.19 ± 0.00 to 0.75 ± 0.30 mU/mg), followed by 1% (0.28 ± 0.20 to 1.45 ± 0.13 mU/mg). Among them, BEE 2% (0.19 ± 0.00 mU/mg) showed higher inhibition of AChE, followed by SO and LEE (0.42 ± 0.03 and 0.75 ± 0.30 mU/mg, respectively), as compared to lower concentrations (0.25–0.5%) (Figure 1a). Similarly, for the GST assay, all the concentrations of LEE, BEE, and SO significantly inhibited the GST activity (F_4,14_ = 31.79 to 500.37, *p* < 0.0001), as compared to the control. Among them, BEE at 2% (5.37 ± 0.50 mU/mg) showed higher inhibition of GST, followed by LEE and SO (9.41 ± 1.13 and 35.01 ± 0.63 mU/mg, respectively), as compared to lower concentrations (0.25–0.5%). However, LEE, BEE, and SO at 2% reported higher inhibition of GST (5.37 ± 0.50 to 35.01 ± 0.63 mU/mg), followed by 1% (7.77 ± 0.11 to 41.26 ± 1.88 mU/mg) (Figure 1b).

## 3. Discussion

The chemical composition of crude fat/oil, *n*-hexane fractions of leaf/bark ethanolic aqueous extracts of *T. sebifera*, isolation of compounds from ethyl acetate and *n*-butanol fractions of leaves, residual toxicity of leaf/bark ethanolic/methanolic extracts, SO and its combinations, fractions, and isolated compounds, and combinations of SO with leaf/bark extracts for their synergistic interaction against *A. craccivora* are discussed. The chemical composition (crude fat/oil) of whole seed/tallow coating/kernel and volatile oils extracted from leaves/stem/flowers of *T. sebifera* by different solvents/fractions vary depending on location/season/species [33,34]. In the present study, *T. sebifera* kernels yielded 50% of the oil, which is higher than an earlier study (LA, USA) where kernels afford 33% of the oil [33], but Chinese tallow tree seeds and tallow coating collected during October 2011 reported 44% and 81% of oil, respectively. In a similar study, castor seeds and jatropha seeds recorded 40–60% and 30–50% of oil, respectively, and these are non-edible oils used for industrial use [35]. Seed oil extracted by *n*-hexane from kernels of *T. sebifera* in the current study contains a higher percentage of unsaturated fatty acids (oleic acid, linoleic acid, and palmitoleic acid) than saturated fatty acids (palmitic and stearic acid). Current results were confirmed with the findings of the previous study, where saturated fatty acids (9.0%) are comparable with the present study, but unsaturated fatty acids (91.4%) are higher [33]. The composition of palmitic acid, linoleic acid, oleic acid, and stearic acid by hexane is comparatively less and palmitoleic acid is higher than in the previous report [33]. In this study, shikimic acid, quercetin-3-o-glucoside, gallic acid, and kaempferol-3-o-glucoside were isolated from ethyl acetate/*n*-butanol fractions. Current results were confirmed with previous studies, where eight phenolic compounds including gallic acid, ellagic acid, hyperin, isoquercitrin, astragalin, quercetin, kaempferol, and rutin were identified from ethyl acetate, *n*-butyl alcohol, and water fractions of *T. sebifera* leaves [10,36].

In the current study, the major metabolites present in the *n*-hexane fraction of the leaves were *n*-hexadecanoic acid, octadecanoic acid, ethyl ester, and neophytadiene, whereas in the bark, the major metabolites were galaxolide, ethyl phthalate, and 1-octadecene. In this study, the volatile composition of leaves/flowers/stem was not studied. However, a previous study reported that the major constituents in the volatile oil from leaves of *T. sebifera* are bis (2-ethylhexyl) phthalate, phytol, and 2,6,10-trimethyl-tetradecane, in the stem are bis (2-ethylhexyl) phthalate and 3-methyl-1-pentanol, and in the flower are bis (2-ethylhexyl) phthalate, 2-methyl-cyclopentanone, and N-phenyl-formamide. The compounds such as bis (2-ethylhexyl) phthalate, 1,2,4,5-tetramethylbenzene, and 2,5-bis (1,1-dimethylethyl)-phenol coexisted in the volatiles of leaves, stem, and flowers [34]. Leaf extract/fractions/seed oil/compounds of *T. sebifera* showed antibacterial, antimicrobial [9], and antioxidant activities, as compared to other solvent extracts [8,9], but these activities were not studied in the current study.

In the present investigation, all the extracts/fractions/compounds/SO of *T. sebifera* showed toxicity against *A. craccivora*. In case of leaf extracts, the LEE 80% was found to be most effective (LC_50_ = 6756.42 mg/L), followed by LME 80%, as compared to other extracts after 96 h of treatment. Results agree with the findings of Ahmed et al. [37], who reported on the ethanolic leaf extract of *Citrullus colocynthis* against *Brevicornye brassicae* (LC_50_ = 10,400 mg/L) after 72 h, but it was not superior to the present study. Similarly, leaf ethanolic extract of *Ricinus communis* was more promising (LC_50_ = 553 mg/L, 58.6% mortality) against *Myzus persicae* [38]. In other studies, leaf methanolic extracts of *Prosopis juliflora* at 1% also showed 86.6% mortality against *A. craccivora* [39]. Similarly, among the bark extracts, BEE 80% (LC_50_ = 5115.98 mg/L) was found to be more effective, followed by BEE 100%, compared to others after 96 h of treatments. The present study also confirms the findings of the previous report, where bark methanol extract of *Prosopis juliflora* at 1% showed 93.3% mortality against *A. craccivora* [39].

The SO of *T. sebifera* in the current study was found to be more effective after 72 and 96 h of treatment (LC_50_ = 2504.59 and 850.94 mg/L, respectively) than leaf and bark extracts. Results agree with the findings of Fenigstein et al. [40], who reported that SO of *Ricinus communis* (1.5%) showed 75% mortality of *Bemisia tabaci*, as compared to the present study where SO of *T. sebifera* recorded higher mortality against aphids at a lower concentration. In a similar study, neem seed oil 2% also showed a 71% reduction in the population of *Lipaphis erysimi* [41]. The insecticidal activity of *T. sebifera* SO in the current study may be due to the presence of unsaturated fatty acids (oleic, linoleic, and palmitoleic acids).

The residual toxicity of the combination of SO with 80% LEE/BEE of *T. sebifera* against *A. craccivora* showed a significant synergistic interaction under laboratory conditions. Among the combinations, BEE+SO (1:1 ratio) was found to be more effective (LC_50_ = 144.26 mg/L) against *A. craccivora*, followed by SO+LEE at a 1:1 ratio, LEE+SO+BEE at a {(1+1):2} ratio, and LEE+BEE at a 1:3 ratio, as compared to other mixtures 96 h after treatment under laboratory conditions. Similarly, under the plant growth chamber, the binary mixture of SO+LEE (LC_50_ = 223.82 mg/L) was more effective against *A. craccivora*, followed by SO+BEE, compared to LEE, SO, and BEE. Based on FEI values, SO+BEE showed a synergistic interaction.

Among the leaf fractions, the *n*-hexane fraction (LC_50_ = 196.61 mg/L) was the most promising against *A. craccivora* as compared to other fractions. However, among the bark fractions, the ethyl acetate fraction was most effective (LC_50_ = 813.45 mg/L) against *A. craccivora*. The efficacy of the *n*-hexane fraction of leaves against *A. craccivora* may be due to the presence of metabolites such as *n*-hexadecanoic acid, galaxolide, ethyl phthalate, octadecanoic acid, ethyl ester, etc. Similarly, in the *n*-hexane fraction of bark, the predominant metabolite is galaxolide, followed by ethyl phthalate and 1-octadecene. It is also confirmed that the efficacy of the tested fractions against *A. craccivora* also may be due to the presence of flavonoids (kaempferol-3-o-glucoside, quercetin-3-o-glucoside) and phenolic acids (gallic acid and shikimic acid). In the current study, the *n*-hexane leaf fraction showed strong efficacy against *A. craccivora* as compared to the *n*-hexane fraction of *Ageratum houstonianum* and *Eupatorium adenophorum* (LC_50_ = 2881–2590 mg/L) after 96 h [42,43]. Present results also confirm the findings of other reports, where the leaf *n*-hexane fraction of *Ricinus communis* showed 92–96% mortality against *Sipha flava* and *Melanaphis sacchari* [44,45]. Similarly, the ethyl acetate leaf fraction in the current study was also reported more promising (LC_50_ = 367.75 mg/L) than the ethyl acetate fraction of *Trillium govanianum* (LC_50_ = 2186.3 mg/L) against *A. craccivora* after 96 h [46].

The residual toxicity of the isolated compounds against *A. craccivora* showed that gallic acid was most effective (LC_50_ = 1303.68 mg/L) against *A. craccivora*, followed by shikimic acid and quercetin-3-o-glucoside, as compared to kaempferol-3-o-glucoside. The present results on the efficacy of gallic acid were confirmed with that of Punia et al. [47], who reported that gallic acid at 3125 mg/L incorporated in a diet fed to the larvae of *S. litura* showed 70% larval mortality, adult emergence, and a delayed developmental period. In the current study, quercetin and kaempferol also showed toxicity against aphids, and these results were also confirmed with findings of earlier studies where kaempferol and quercetin from methanolic extract of *Buddleja albiflora* Hemsl. showed efficacy against *P. xylostella* (LC_50_ = 1.454 mg/mL) and *Mythimna separata* (74–77% mortality), and antifeedant activity against *P. xylostella* (AFC_50_ = 1.019 mg/mL) [48].

Detoxification enzymes such as GST, cytochrome P450 monooxygenases, and carboxyl/cholinesterases are well-known for helping plant-feeding insects to maintain their physiological roles by detoxifying xenobiotic substances [49,50]. Pesticides and poisonous secondary metabolites from host plants are examples of xenobiotic substances [35]. The plant extracts/fractions/essential oils have a variety of modes of action against insects, including neurotoxicity, insect growth/digestive enzyme inhibition, and inhibition of GST and cytochrome P450 monooxygenase [51]. The AChE activities in *A. craccivora* fed with bean leaf discs treated with different concentrations of LEE, BEE, and SO showed significant inhibition compared to control aphids. Present results were confirmed with the findings of Dolma et al. [52], who reported that *Tagetes minuta* oil showed an inhibitory effect on AChE in *P. xylostella*. In a similar study, essential oils and synthesized nano-emulsions from *Basilicum ocimum* L., *Cuminum cyminum* L., *Origanum marjorana* L., and *Matricaria chamomilla* L. showed decreased activity of AChE in *A. craccivora* [53]. Similarly, for the GST assay, all the concentrations of LEE, BEE, and SO significantly inhibited the GST activity as compared to the control. Results conform with the findings of Phankaen et al. [54], who reported that caffeine from *Coffea arabica* extract inhibits GST and carboxylesterase (CarE) in *Tribolium castaneum*. Similarly, a previous study also showed that *Tagetes minuta* oil inhibited GST in *P. xylostella* [52].

The current study concludes that extracts from leaves and bark, fractions, and isolated compounds of *T. sebifera* can be used for the control of *A. craccivora*. The insecticidal activity might be due to the presence of individual or combined action of extracts and SO of *T. sebifera*. Based on the literature survey, the efficacy of *T. sebifera* extracts/fractions/isolated compounds and SO against *A. craccivora*, as well as the chemical composition of SO and *n*-hexane fractions, had not previously been reported. Thus, this study is unique and has been executed for the first time to examine the leaf and bark extracts and SO of *T. sebifera* against *A. craccivora*.

## 4. Materials and Methods

### 4.1. Plant Material

The leaves at the vegetative stage, bark, and seeds of *T. sebifera* used in the study were collected in and around CSIR-IHBT Campus, Palampur, Himachal Pradesh (32°06′05″ N, 76°34′10″ E), during May to September 2020 located in the Dhauladhar range of the western Himalayas. The authentication of plant material was performed by a Taxonomist of CSIR-IHBT, Palampur, India, and voucher specimens were deposited in the herbarium (voucher No. PLP 18563). The plant material was dried under shade for 15 days and was used for further processing.

### 4.2. Preparation of Leaf/Bark Extracts and Fractions

The well-dried leaves and bark samples were powdered using a grinder. About 1 kg of samples was extracted separately by maceration (3 × 5 L) in 12 h intervals using a concentration of 50%, 80%, and 100% ethanol:water at room temperature. The macerated samples were filtered, and then the solvent was removed under low pressure at 45 °C in a rotatory evaporator (Buchi, R-210); accordingly, leaf/bark ethanolic/methanolic extracts at a concentration of 50%, 80%, and 100% were also prepared. The yields obtained for 50%, 80%, and 100% leaf ethanolic aqueous extracts (LEE) were 78.8, 90.5, and 51.4 g, respectively. Similarly, the yields obtained for 50%, 80%, and 100% leaf methanolic aqueous extracts (LME) were 77.3, 72.6, and 48.6 g, respectively. The yields obtained for 50%, 80%, and 100% bark ethanolic aqueous extracts (BEE) were 77.5, 85.6, and 53.2 g, respectively. In the same way, the yields obtained for 50%, 80%, and 100% bark methanolic aqueous extracts (BME) were 67.5, 78.5, and 63.3 g, respectively. All the extracts were kept at <4 °C until further analysis.

**Figure 2:**
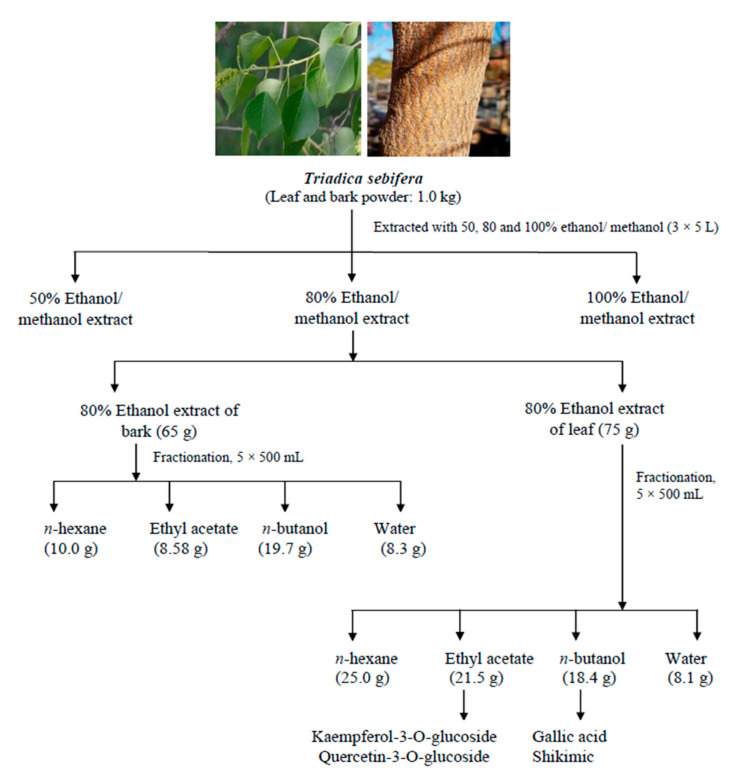
Schematic representation of extraction and fractionation of *Triadica sebifera*.

The dried ethanolic aqueous extract 80% from leaves (75 g) and bark (65 g) was dissolved in a minimum volume of distilled water and sequentially fractionated (500 mL × 5) from lower to higher polarity of the solvents (*n*-hexane, ethyl acetate, *n*-butanol, and water) and evaporated separately at 45 °C using a rotary evaporator and then lyophilized to obtain the respective fractions. The yields obtained for the *n*-hexane, ethyl acetate, *n*-butanol, and water fractions of leaves were 25, 21.5, 18.4, and 8.1 g, respectively. Similarly, the yields obtained for the *n*-hexane, ethyl acetate, *n*-butanol, and water fractions of bark were 10, 8.58, 19.7, and 8.3 g, respectively. All the fractions were kept at <4 °C until further analysis (Figure 2).

### 4.3. Extraction of SO

About 10 kg of seeds were dried under the shade for 15 days. The seed coat was stripped manually, and defatted by mixing them in hot water (70 °C) using a mini extractor. The defatting process was regulated in the mini extraction system for 3.5 h/percolation 6 times for the appearance of black kernels, and then cleaned with hot water and transferred to a hot-air oven (45 °C) for drying. When the kernels were dehydrated, 2 kg of kernels were grounded in a blender and dissolved in *n*-hexane (3 L × 4 times) in a percolator. The eluted solvent was collected, filtered, and removed under low pressure at 45 °C in Rotavapor (Buchi, R-210), which yielded 1 L of SO which was kept at <4 °C until further analysis.

### 4.4. Isolation of Compounds

The ethyl acetate fraction (8 g) was taken and subjected to column chromatography over 60–120-mesh size silica gel. The column was packed in pure chloroform and eluted with methanol in the increasing order of polarity. A total of 50 fractions were collected (100 mL each) using a gradient elution of chloroform and methanol mixture. All the fractions were then divided into seven main fractions (i.e., F_A_–F_G_) after TLC analyses. Fractions F_C_–F_D_ were eluted at polarity (20:80) MeOH:CHCl_3_ and then again subjected for column chromatography over 60–120-mesh size silica gel, resulting in the isolation of compound **1** (128 mg) at polarity (10:90) MeOH:CHCl_3_ and compound **2** (150 mg) at polarity (15:85) MeOH:CHCl_3_.

The *n*-butanol fraction (6 g) was subjected to column chromatography for isolation of pure molecules. Mesh size 60–120 silica was used with the solvent system chloroform:methanol:formic acid for column chromatography. At different polarities and TLC-based analysis, five major fractions (i.e., BF_A_–BF_E_) were collected. BF_A_ fraction was again subjected to 230–400 silica for isolation. The solvent system used for isolation was chloroform:methanol with 0.01% formic acid. Compound **3** (124 mg) was isolated at 3% methanol:chloroform with 0.01% formic acid, and compound **4** (108 mg) was isolated at 5% methanol:chloroform with 0.01% formic acid.

### 4.5. Preparation of Fatty Acid Methyl Esters (FAMES)

The FAMES were prepared as per the method followed by Adebisi et al. [43]. Briefly, 1 mL of *n*-hexane was added into 0.1 mL of oil and 1 mL of sodium methoxide (1.5 g of NaOH in 50 mL of methanol). The mixture was stirred vigorously using a vortex and kept untouched for 10 min to segregate the transparent fatty acid methyl ester solution from the opaque aqueous layer, and was stored at 4 °C until further use.

### 4.6. Gas Chromatography-Mass Spectrophotometry (GC-MS) Analysis

The GC-MS analysis of *n*-hexane fractions of leaf and bark ethanolic aqueous extracts and SO were carried out using a Shimadzu QP 2010 and DB–5MS (J & W Scientific, Folsom, CA, USA) capillary column (30 m × 0.25 mm, i.d., 0.25 μm thickness). The GC oven temperature was programmed at 70 °C for 4 min and then increased to 220 °C at 4 °C/min and held for 5 min. The injector temperature was 240 °C, interface temperature was 250 °C, acquisition mass range was 800–50 amu, and ionization energy was 70 eV. Helium was used as a carrier gas. Compounds were identified using a library search of the National Institute of Standards and Technology (NIST) database [55] and mass spectral fragmentation patterns with those reported in the literature [56].

### 4.7. Test Insect

*A. craccivora* was collected from the open-field conditions and maintained/reared on live French bean, *Phaseolus vulgaris* L., under controlled conditions (25 ± 2 °C temperature, 60% ± 5% humidity, and photoperiod of 16 h light and 8 h dark) for more than 100 generations. The fresh nymphs of 3–4 days old were selected for experiments.

### 4.8. Preliminary Screening

Preliminary screening of leaf/bark extracts, fractions, SO, and compounds was carried out at higher concentrations (5000–10,000 mg/L) for their efficacy against nymphs of *A. craccivora* [46,57]. Based on preliminary data, 5–6 concentrations were prepared and evaluated against aphids in the final bioassay experiments.

### 4.9. Residual Toxicity of Leaf/Bark Aqueous Ethanolic/Methanolic Extracts under Laboratory Conditions

Residual toxicity of leaf/bark extract/fractions and SO was tested against nymphs of *A. craccivora* by the Potter spray method as per the standard method [58]. Briefly, five concentrations of LEE/LME/BEE/BME (500–20,000 mg/L), leaf and bark fractions (250–10,000 mg/L), SO (500–10,000 mg/L), and compounds (312.5–5000 mg/L) were prepared. The leaf discs of 3 cm-diameter were prepared and pressed over the water-agar medium on Petri plates to maintain the freshness of leaf discs. About 10 nymphs/leaf disc were released in each Petri plate. Each treatment was replicated three times. The test solution (2 mL) was sprayed under Potter’s spray tower operated at 1.1 kg/cm^2^ pressure and incubated under controlled conditions. Observations on dead insects were recorded at 24 h intervals and up to 96 h. The commercial neem-based formulation, i.e., Indo-neem (mixture of Azadirachtin 0.15% EC and neem oil 35%, manufactured by Pest Control India Pvt. Limited, Goa, India), was used as a positive control for comparison.

### 4.10. Residual Toxicity of Binary Mixtures of Leaf/Bark Extracts and SO under Laboratory Conditions

Based on a preliminary study, the blends/mixtures of SO with leaf/bark ethanol aqueous extract of *T. sebifera* were prepared in five concentrations (62.5–1000 mg/L) and proportions, as 1:3 (SO: LEE), 1:1 (SO: LEE), 3:1 (SO: LEE), 1:1:1 (SO: LEE: BEE), {(1+1): 2} (LEE+SO: BEE), {(1+1): 2} (LEE+BEE: SO), and (1+1): 2 (SO+BEE: LEE), for the bioassay and synergistic activity against *A. craccivora* under laboratory conditions. Five concentrations (62.5–1000 mg/L) were prepared and sprayed against nymphs of *A. craccivora* as described above. Each treatment was replicated three times. Observations on mortality were recorded at 24, 48, 72, and 96 h after treatment. The fractional effect indices (FEI) were calculated to study the joint action of binary mixtures/combinations. FEI = fractional effect of A + fractional effect of B, in which fractional effect of A = LC_50_ mixture/LC_50_ of A and fractional effect of B = LC_50_ mixture/LC_50_ of B [59]. The FEIs were interpreted based on Bassole et al.’s [60] classifications as being synergistic if FEI < 0.5, additive if FEI ≥ 0.5 and ≤ 1.0, indifferent if FEI > 1.0 and ≤ 4.0, or antagonistic if FEI > 4.0.

### 4.11. Residual Toxicity of Leaf/Bark Extracts and Their Binary Mixtures under Plant Growth Chamber

The residual toxicity of leaf/bark ethanolic aqueous extract, SO at six concentrations (625–20,000 mg/L), and their combinations of *T. sebifera* (SO+LEE, SO+BEE) in a 1:1 ratio was prepared in five concentrations (125–2000 mg/L) and evaluated against *A. craccivora* under controlled conditions (26 ± 2 °C, 70% ± 5% relative humidity, and photoperiod of 16 h light and 8 h dark) in a plant growth chamber (Percival Scientific, Perry, IA, USA). The *Phaseolus vulgaris* plants were raised in plastic pots (7 × 9 cm) filled with the potting mixture (1:1:1 ratio of vermiculate:cocopeat:perlite). One-week-old plants (3–4 leaf stage) were inoculated with two-day-old nymphs of *A. craccivora* and allowed to settle for 24 h. After settling, test solutions/concentrations were sprayed on plants using a hand sprayer. Observations of the number of dead insects/plants were recorded at 24, 48, 72, and 96 h after treatment. There were six treatments, and each treatment was replicated five times. The fractional effect indices (FEI) were calculated as mentioned above to study the joint action of binary mixtures/combinations.

### 4.12. Enzyme Inhibition Activities of LEE, BEE, and SO against A. craccivora

Four different concentrations of LEE, BEE, and SO (0.25%, 0.5%, 1%, and 2%) were chosen for enzyme activity [52] according to the residual toxicity assay described above. The nymphs of *A. craccivora* of each concentration after 48 h of treatment were homogenized and centrifuged at 15,000 rpm at 4 °C for 30 min. The pellet was discarded, and the supernatant was used for the enzyme activity. Before proceeding with enzyme activity, the total protein concentration was measured using the Bradford reagent. For the enzyme assay of glutathione S-transferase (GST) and acetylcholine esterase (AChE), the assay kits were procured from Cayman Chemicals (Ann Arbor, MI, USA) and Abcam (Cambridge, UK), respectively.

### 4.13. Data Analysis

The mortality data of aphids based on the residual toxicity of extracts, fractions, SO, combinations, and pure compounds were compiled. The median lethal concentration values (LC_50_) and other regression parameters were determined by Probit [61] using SPSS 10 software, version 16. The FEI for binary mixtures/combinations were calculated to study the joint action studies (synergistic, additive, indifferent, and antagonistic). Similarly, percent mortality data against aphids were also analyzed by one-way analysis of variance, and means were compared by Tukey’s post hoc test.

## 5. Conclusions

The leaf/bark extracts BEE 80% and 100% were found to be the most effective, followed by BME 50%, as compared to LEE and LME 80%. The SO was found to be more effective than leaf and bark extracts. Among combinations, BEE+SO (1:1 ratios) was the most effective against *A. craccivora*, followed by SO+LEE at a 1:1 ratio, LEE+SO+BEE at a (1+1):2 ratio, and LEE+BEE at a 1:3 ratio. All the combinations/blends showed synergistic activity to *A. craccivora*, but LEE+BEE at 1:3, 3:1, and 1:1 ratios showed the most significant synergistic interaction. Among leaf/bark fractions, the *n*-hexane leaf fraction was the most effective against aphids. Among pure compounds, gallic acid was found to be the most effective. LEE, BEE, and SO significantly inhibited the AChE and GST activity in *A. craccivora*. Based on our findings, the leaf extracts/SO and their combinations can be recommended for the control of aphids in crop plants under greenhouse/field conditions.

## Figures and Tables

**Table 1:** Chemical composition of fatty acid methyl esters present in SO of *Triadica sebifera*.

Sr. No.	Compound’s Name	Area (%)	RI^a^ *	RI^b^ **	Mass Fragmentation
1	9-hexadecenoic acid, methyl ester (Palmitoleic acid, C_6_: 1)	2.13	1933	1931 [13]	268 [M^+^], 236, 207, 194, 152, 138, 123, 97, 74, 69, 55, 41
2	Hexadecanoic acid, methyl ester (Palmitic acid, C_16_: 0)	4.99	1986	1984 [14]	270 [M^+^], 239, 227, 199, 185, 171, 143, 129, 101, 87, 74, 57, 41
3	9,12-octadecadienoic acid (Z, Z), methyl ester (Linoleic acid, C_18_: 2)	15.42	2092	2094 [15]	294 [M^+^], 262, 178, 164, 150, 135, 123, 109, 95, 81, 67, 55, 41
4	9-octadecenoic acid, methyl ester (Oleic acid, C_18_: 1)	8.78	2100	2101 [16]	296 [M^+^], 264, 222, 180, 166, 152, 137, 123, 97, 83, 69, 55, 41
5	Octadecanoic acid, methyl ester (Stearic acid, C_18_: 0)	1.38	2121	2128 [17]	298 [M^+^], 255, 213, 199, 143, 129, 101, 87, 74, 57, 43, 41

* RI^a^ = Calculated Retention Indices; ** RI^b^ = Retention Indices from the literature.

**Table 2:** Chemical composition of *n*-hexane fraction from leaves and bark of *Triadica sebifera*.

Metabolites	Molecular Formula	Area (%)	RI^a^ *	RI^b^ **	Mass Fragmentation
**Leaf**					
1,8-cineole	C_10_H_18_O	1.61	1037	1037 [23]	*m*/*z* 154 (M^+^), 139, 125,108, 84, 81, 69, 43, 41
Fenchyl acetate	C_12_H_20_O_2_	0.70	1230	1223 [24]	*m*/*z* 154 (M^+^), 136, 121, 108, 95, 80, 69, 43, 41, 27
Neophytadiene	C_20_H_38_	5.87	1841	1836 [25]	*m*/*z* 137 (M^+^), 123, 109, 95, 82, 68, 43, 41
*n*-hexadecanoic acid	C_16_H_32_O_2_	15.61	1961	1962 [26]	*m*/*z* 256 (M^+^), 227, 213, 199, 185, 171, 157, 143, 129, 115, 98, 85, 73, 60, 43, 41
cis, cis, cis-7,10,13-hexadecatrienal	C_16_H_26_O	3.06	1992	1989 [27]	*m*/*z* 264 (M^+^), 222, 149, 135, 121, 108, 95, 79, 67, 55, 41
Octadecanoic acid, ethyl ester	C_20_H_40_O_2_	9.85	2189	2189 [28]	*m*/*z* 312 (M^+^), 269, 213, 157, 143, 129, 115, 101, 88, 70, 57, 43, 41
**Bark**					
Hexanoic acid	C_6_H_12_O_2_	0.31	984	989 [28]	*m*/*z* 87 (M^+^), 73, 60, 43, 41, 40
2-decenal	C_10_H_18_O	0.23	1263	1260 [29]	*m*/*z* 121 (M^+^), 98, 84, 70, 57, 43, 41, 40
Ethyl phthalate	C_12_H_17_O_4_	28.43	1587	1585 [30]	*m*/*z* 222 (M^+^), 177, 149, 121, 105, 93, 76, 65, 50
1-octadecene	C_18_H_36_	2.69	1803	1800 [31]	*m*/*z* 125 (M^+^), 111, 97, 83, 57, 41, 40
Galaxolide	C_18_H_26_O	44.73	1834	1837 [32]	*m*/*z* 258 (M^+^), 243, 213, 185, 171, 157, 143, 128

* RI^a^ = Calculated Retention Indices; ** RI^b^ = Retention Indices from the literature.

**Table 3:** Efficacy of ethanolic/methanolic leaf/bark aqueous extracts and SO of *Triadica sebifera* against *Aphis craccivora*.

Leaf Extracts	LC_50_ * (mg/L)	Confidence Limits (mg/L)	Slope ± SE	Chi Square	*p*-Value
LEE 100% (72 h)	14,100.0	11,764.41–17,527.10	2.31 ± 0.53	0.36	0.99
LEE 100% (96 h)	8702.07	6880.35–10,137.00	3.08 ± 0.52	0.61	0.99
LEE 80% (72 h)	9590.49	7706.54–11,128.43	2.91 ± 0.52	1.81	0.87
LEE 80% (96 h)	6756.42	5342.84–7885.95	3.97 ± 0.58	1.84	0.87
LEE 50% (72 h)	38,860.0	24,892.46–341,196.32	2.07 ± 0.72	0.67	0.98
LEE 50% (96 h)	28,570.0	21,383.79–71,689.83	2.51 ± 0.71	0.68	0.98
LME 100% (72 h)	9627.0	6881.53–11,725.61	2.07 ± 0.49	2.02	0.85
LME 100% (96 h)	7528.56	5691.95–8931.12	3.04 ± 0.52	5.07	0.41
LME 80% (72 h)	10,800.0	8603.89–12,754.79	2.45 ± 0.50	2.24	0.81
LME 80% (96 h)	7120.27	5593.04–8326.45	3.63 ± 0.55	6.95	0.22
LME 50% (72 h)	11,540.0	9689.45–13,331.76	2.86 ± 0.53	3.76	0.58
LME 50% (96 h)	7579.55	6239.59–8676.68	4.15 ± 0.58	5.80	0.33
SO (72 h)	2504.59	1675.92–3562.91	1.18 ± 0.21	0.86	0.97
SO (96 h)	850.938	533.52–1171.05	1.69 ± 0.25	3.28	0.66
**Bark extracts**					
BEE 100% (72 h)	8325.46	6958.41–9455.76	4.05 ± 0.57	7.39	0.19
BEE 100% (96 h)	5228.89	4038.43–6165.80	4.88 ± 0.78	3.83	0.57
BEE 80% (72 h)	7300.57	5889.44–8435.86	3.97 ± 0.57	6.17	0.29
BEE 80% (96 h)	5115.98	3613.44–6219.77	4.04 ± 0.75	1.31	0.73
BEE 50% (72 h)	10,650.0	8244.02–12,743.89	2.25 ± 0.50	3.73	0.59
BEE 50% (96 h)	7098.41	5159.65–8546.32	2.91 ± 0.52	7.32	0.20
BME 100% (72 h)	8737.64	6586.26–10,377.96	2.61 ± 0.50	2.12	0.83
BME 100% (96 h)	5701.69	4147.42–6880.23	3.73 ± 0.63	2.34	0.67
BME 80% (72 h)	9490.58	7504.78–11,085.89	2.78 ± 0.51	0.45	0.99
BME 80% (96 h)	5779.72	4187.11–7007.96	3.55 ± 0.58	1.42	0.92
BME 50% (72 h)	10,580.0	8431.88–12,452.44	2.51 ± 0.51	1.43	0.92
BME 50% (96 h)	5233.81	2941.71–6869.58	2.51 ± 0.53	7.29	0.20
Azadirachtin (72 h)	2642.32	2013.70–3816.64	1.53 ± 0.22	0.99	0.80
Azadirachtin (96 h)	1174.22	973.61–1416.60	2.28 ± 0.25	5.16	0.16

* LC_50_ = Lethal concentration to kill 50% of test insect; Mean of three replications; *n* = 150 insects per treatment; LC_50_ was calculated for fractions showing >50% mortality using Probit analysis.

**Table 4:** Efficacy of fractions of *Triadica sebifera* leaves and bark against *Aphis craccivora*.

Leaf Fractions	LC_50_ (mg/L) *	Confidence Limits (mg/L)	Slope ± SE	Chi Square	*p*-Value
*n*-hexane (72 h)	425.73	196.38–679.40	1.09 ± 0.21	1.55	0.82
*n*-hexane (96 h)	196.61	76.21–316.54	1.57 ± 0.33	2.45	0.65
Ethyl acetate (72 h)	838.89	558.64–1178.77	1.39 ± 0.21	3.96	0.41
Ethyl acetate (96 h)	367.75	230.65–503.57	1.88 ± 0.32	0.75	0.94
*n*-butanol (72 h)	1527.84	1123.04–2093.81	1.60 ± 0.22	1.14	0.89
*n*-butanol (96 h)	990.22	746.43–1294.96	1.92 ± 0.25	2.68	0.61
Water (72 h)	2702.82	1799.12–4556.10	1.12 ± 0.19	1.34	0.85
Water (96 h)	864.68	643.83–1133.25	1.89 ± 0.25	2.07	0.72
**Bark fractions**					
*n*-hexane (72 h)	1659.98	1211.70–2310.56	1.54 ± 0.21	3.62	0.46
*n*-hexane (96 h)	1130.95	867.74–1467.86	2.02 ± 0.26	1.95	0.75
Ethyl acetate (72 h)	3629.52	2322.33–6984.03	1.05 ± 0.19	3.60	0.46
Ethyl acetate (96 h)	813.45	613.57–1052.33	2.04 ± 0.27	0.80	0.94
*n*-butanol (72 h)	3539.63	2343.17–6296.58	1.15 ± 0.20	1.92	0.75
*n*-butanol (96 h)	1071.81	762.69–1472.81	1.52 ± 0.22	5.26	0.26
Water 72 h)	3049.50	2087.70–4995.85	1.24 ± 0.20	5.94	0.20
Water (96 h)	915.15	684.74–1198.41	1.90 ± 0.25	2.55	0.63
Azadirachtin (72 h)	2642.32	2013.70–3816.64	1.53 ± 0.22	0.99	0.80
Azadirachtin (96 h)	1174.22	973.61–1416.60	2.28 ± 0.25	5.16	0.16

* LC_50_ = Lethal concentration to kill 50% of test insect; Mean of three replications; *n* = 150 insects per treatment; LC_50_ was calculated for fractions showing >50% mortality using Probit analysis.

**Table 5:** Efficacy of compounds isolated from leaf fractions of *Triadica sebifera* against *Aphis craccivora*.

Compounds	LC_50_ (mg/L) *	Confidence Limits (mg/L)	Slope ± SE	Chi Square	*p*-Value
Kaempferol-3-O-glucoside (72 h)	4512.54	3455.14–6789.22	2.02 ± 0.31	0.94	0.82
Kaempferol-3-O-glucoside (96 h)	3762.69	2924.95–5398.97	1.95 ± 0.28	1.36	0.72
Quercetin-3-O-glucoside (72 h)	3068.62	2462.99–4093.71	2.09 ± 0.28	2.21	0.53
Quercetin-3-O-glucoside (96 h)	1855.93	1550.48–2261.15	2.39 ± 0.27	2.60	0.46
Gallic acid (72 h)	2339.69	1928.91–2933.01	2.24 ± 0.27	2.03	0.57
Gallic acid (96 h)	1303.68	1118.34–1520.76	3.07 ± 0.32	1.44	0.70
Shikimic acid (72 h)	2826.31	2320.31–3606.86	2.30 ± 0.29	1.68	0.64
Shikimic acid (96 h)	1725.09	1447.64–2080.49	2.47 ± 0.27	0.23	0.97
Azadirachtin (72 h)	2642.32	2013.70–3816.64	1.53 ± 0.22	0.99	0.80
Azadirachtin (96 h)	1174.22	973.61–1416.60	2.28 ± 0.25	5.16	0.16

* LC_50_ = Lethal concentration to kill 50% of test insect; Mean of five replications; *n* = 300 insects per treatment; LC_50_ was calculated for fractions showing >50% mortality using Probit analysis.

## Data Availability

Not applicable.

## Supplementary Materials

The following supporting information can be downloaded at: https://www.mdpi.com/article/10.3390/molecules27061967/s1, Figure S1: ESI-MS of kaempferol-3-o-glucoside. Figure S2: Chemical structures of isolated compounds from *Triadica sebifera* from ethyl acetate and *n*-butanol fractions of the leaves: (a) kaempferol-3-o-glucoside, (b) quercetin-3-o-glucoside, (c) gallic acid, (d) shikimic acid. Figure S3: ^1^H-NMR spectrum of kaempferol-3-o-glucoside. Figure S4: ^13^C NMR spectrum of kaempferol-3-o-glucoside. Table S1: Observed data and reported data of kaempferol-3-o-glucoside and quercetin 3-o-glucoside from *Triadica sebifera*. Figure S5: ESI-MS of quercetin-3-o-glucoside. Figure S6: ^1^H NMR spectrum of quercetin-3-o-glucoside. Figure S7: ^13^C NMR spectrum of quercetin-3-o-glucoside. Figure S8: ESI-MS of gallic acid. Figure S9: ^1^H NMR spectrum of gallic acid. Figure S10: ^13^C NMR spectrum of gallic acid. Table S2: Observed data and reported data of gallic acid and shikimic acid from *Triadica sebifera*. Figure S11: ESI-MS of shikimic acid. Figure S12: ^1^H NMR spectrum of shikimic acid. Figure S13: ^13^C NMR spectrum of shikimic acid. Table S3: Synergistic activity of leaf/bark extracts and SO of *Triadica sebifera* against *Aphis craccivora* (72 h). Table S4: Synergistic activity of leaf/bark extracts and SO of *Triadica sebifera* against *Aphis craccivora* (96 h). Table S5: Efficacy of leaf/bark ethanolic aqueous extracts and SO of *Triadica sebifera* against *Aphis craccivora* under plant growth chamber. Figure S14: Bio-efficacy of compounds isolated from leaf fractions of *Triadica sebifera* against *Aphis craccivora*.

## Sample Availability

Not available.
