# Supplementary material for: Insecticidal and Enzyme Inhibition Activities of Leaf/Bark Extracts, Fractions, Seed Oil and Isolated Compounds from Triadica sebifera (L.) Small against Aphis craccivora Koch
Source: Molecules. 2022 Mar 18;27(6):1967. doi: 10.3390/molecules27061967 (PMC8954943; doi:10.3390/molecules27061967)
Supplement: Supplementary file 1 [file molecules-27-01967-s001.zip › molecules-1588516-supplementary.pdf]

Supplementary Material

Insecticidal and enzyme inhibition activities of leaf/bark extracts, fractions, seed oil and isolated compounds from *Triadica sebifera* (L.) Small against *Aphis craccivora* Koch

Shudh Kirti Dolma[1,2], Prithvi Pal Singh[2,3] and Sajjalavarahalli G. Eswara Reddy[1,2*]

[1]Entomology Laboratory, Agrotechnology Division, CSIR-Institute of Himalayan Bioresource Technology, Palampur 176061, India; skdolma@gmail.com (S.K.D.)

[2]Academy of Scientific and Innovative Research (AcSIR), Ghaziabad 201002, India

[3]Chemical Technology Division, CSIR-Institute of Himalayan Bioresource Technology, Palampur 176061, India; thakurprithvi028@gmail.com (P.P.S.)

*Corresponding: ereddy@ihbt.res.in, ereddy2001@yahoo.com (S.G.E. Reddy)

Contents

Fig. S1. ESI-MS of kaempferol-3-O-glucoside

Fig. S2. Chemical structures of isolated compounds from *Triadica sebifera* from ethyl acetate and *n*-butanol fractions of the leaf **a.** Kaempferol-3-O-glucoside **b.** Quercetin-3-O-glucoside **c.** Gallic acid **d.** Shikimic acid

Fig. S3. ^{1}H-NMR spectrum of kaempferol-3-O-glucoside

Fig. S4. ^{13}C NMR spectrum of kaempferol-3-O-glucoside

Table S1. Observed data and reported data of kaempferol-3-O-glucoside and quercetin 3-O-glucoside from *Triadica sebifera*

Fig. S5. ESI-MS of quercetin-3-O-glucoside

Fig. S6. ^{1}H NMR spectrum of quercetin-3-O-glucoside

Fig. S7. ^{13}C NMR spectrum of quercetin-3-O-glucoside

Fig. S8. ESI-MS of gallic acid

Fig. S9. ^{1}H NMR spectrum of gallic acid

Fig. S10. ^{13}C NMR spectrum of gallic acid

Table S2. Observed data and reported data of gallic acid and shikimic acid from *Triadica sebifera*

Fig. S11. ESI-MS of shikimic acid

Fig. S12. ^{1}H NMR spectrum of shikimic acid

Fig. S13. ^{13}C NMR spectrum of shikimic acid

Table S3. Synergistic activity of leaf/bark extracts and seed oil of *Triadica sebifera* against *Aphis craccivora* (72 h)

Table S4. Synergistic activity of leaf/bark extracts and seed oil of *Triadica sebifera* against *Aphis craccivora* (96 h)

Table S5. Efficacy of leaf/bark ethanolic aqueous extracts and seed oil of *Triadica sebifera* against *Aphis craccivora* under plant growth chamber

Fig. S14. Bio-efficacy of compounds isolated from leaf fractions of *Triadica sebifera* against *Aphis craccivora*; Figures in the same alphabetical letters in the error bars are statistically at par by Tukey's HSD (P ≥ 0.05)

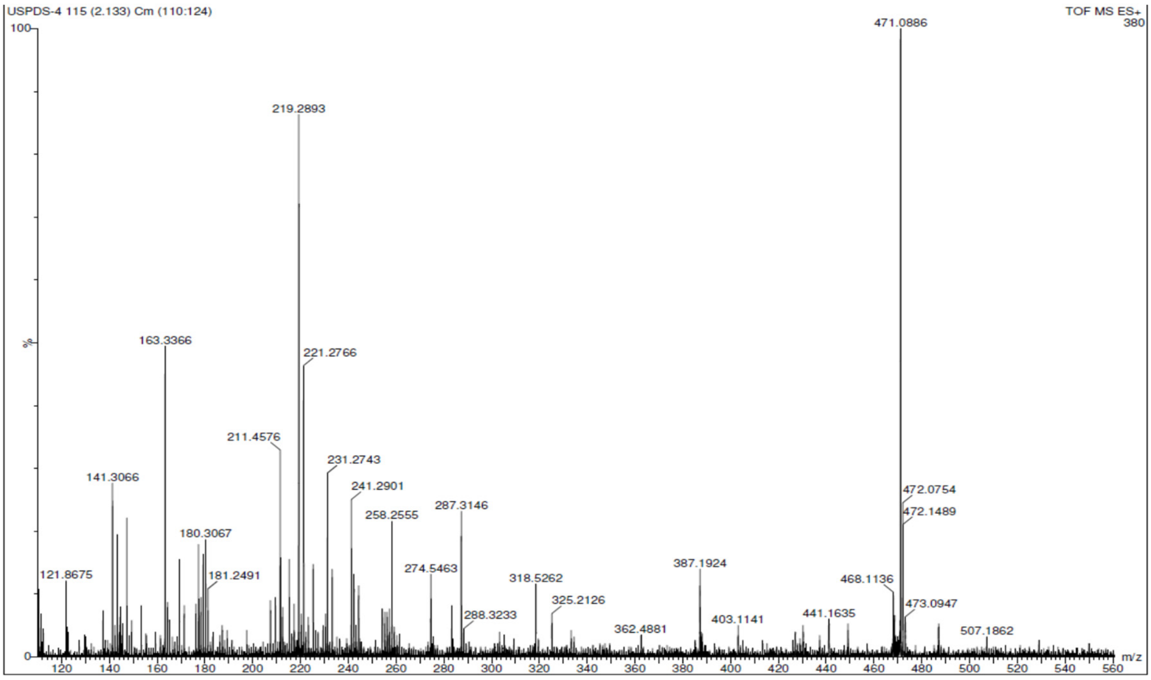

Fig. S1. ESI-MS of kaempferol-3-O-glucoside

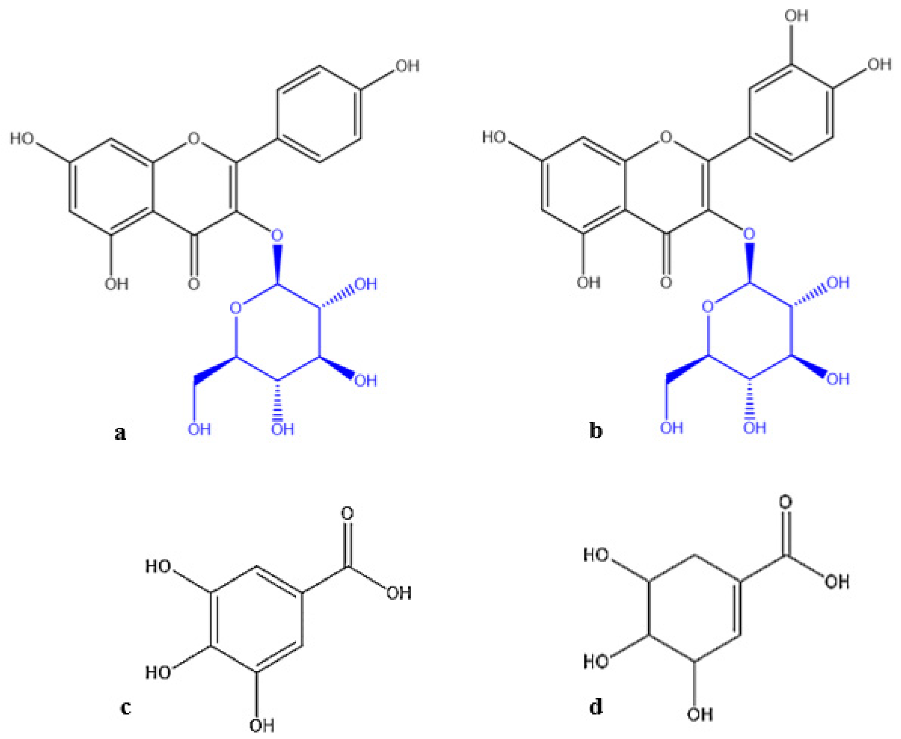

Fig. S2. Chemical structures of isolated compounds from *Triadica sebifera* from ethyl acetate and n-butanol fractions of the leaf **a.** Kaempferol-3-O-glucoside **b.** Quercetin-3-O-glucoside **c.** Gallic acid **d.** Shikimic acid

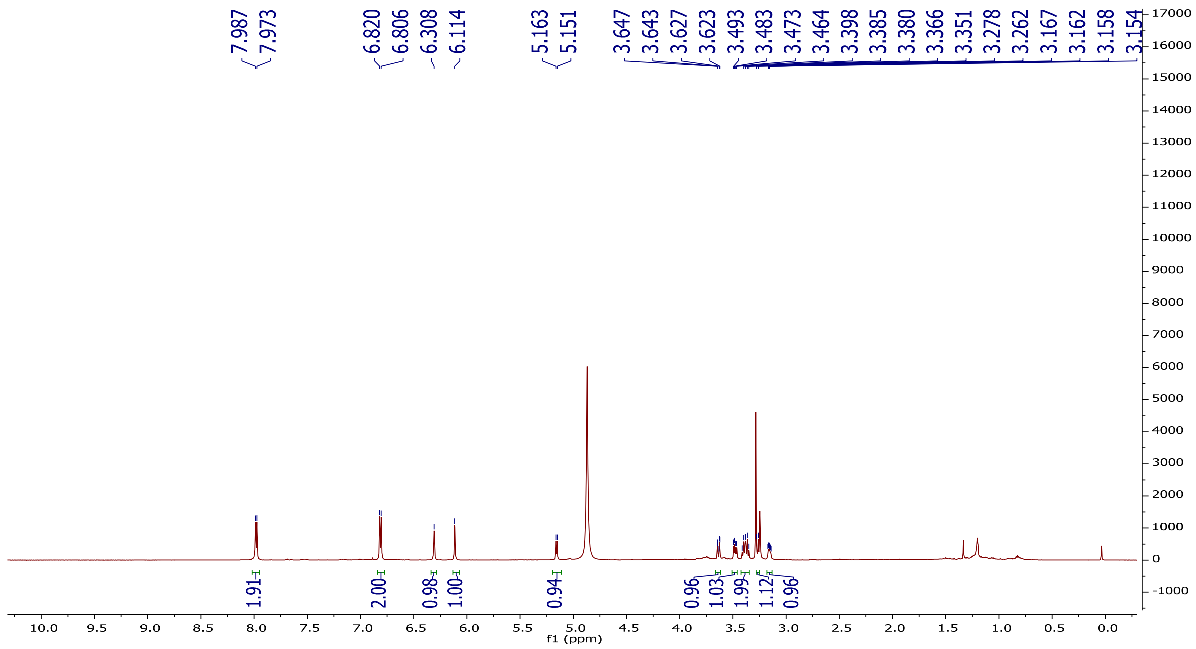

Fig. S3. ^{1}H-NMR spectrum of kaempferol-3-O-glucoside

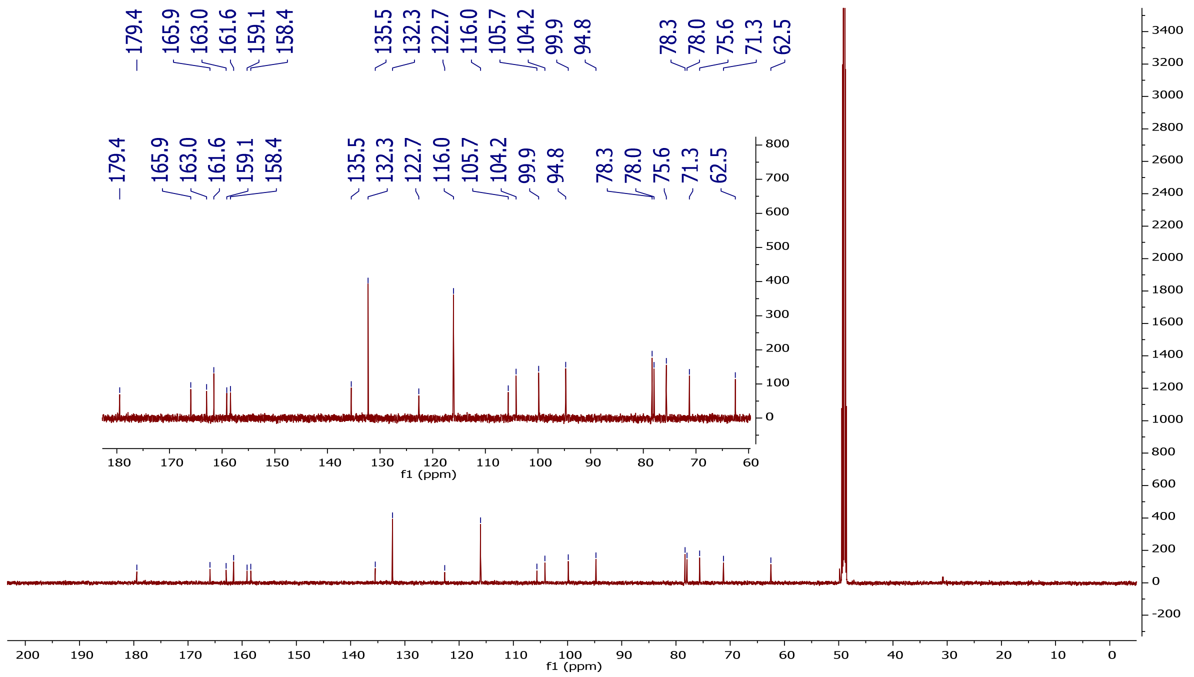

Fig. S4. ^{13}C NMR spectrum of kaempferol-3-O-glucoside

Kaempferol-3-O-glucoside					Quercetin 3-O-glucoside			
Observed data			Reported data		Observed data		Reported data	
Position	δC	δH	δC	δH	δC	δH	δC	δH
1	-	-	-	-		-		-
2	159.1	-	157.6	-	159.1	-	159.3	-
3	135.5	-	134.9	-	135.6	-	135.6	-
4	179.4	-	177.9	-	179.5	-	179.6	-
4a	163.0	-	161.3	-	163.0	-	162.6	-
5	99.9	6.11, s	99.2	6.2	100.0	6.15 d, (*J*= 2.4Hz)	100.2	6.02 s
6	165.9	-	164.9	-	166.3	-	165.7	-
7	94.8	6.30, s	94.3	6.4	94.8	6.34 d, (*J*= 2.4 Hz)	95.9	6.21 s
8	158.4	-	157.0	-	158.5	-	158.4	-
8a	105.7	-	104.3	-	105.6	-	105.8	-
1′	122.7	-	121.0	-	123.1	-	123.2	-
2′	132.3	7.98, d (*J*= 8.4 Hz)	131.0	8.06	117.6	7.66 d, (*J*= 1.8 Hz)	116.5	7.59 s
3′	116.0	6.81, d (*J*= 8.4 Hz)	115.7	6.9	145.9	-	145.7	-
4′	161.6	-	160.1	-	149.9	-	149.8	-
5′	116.0	6.81, d (*J*= 8.4 Hz)	115.7	6.9	116.0	6.82 d, (*J*= 8.4 Hz)	117.8	6.71 d, (*J*= 8.1 Hz)
6′	132.3	7.98, d (*J*= 8.4 Hz)	131.0	8.06	123.2	7.54 dd, (*J*= 8.4,1.8 Hz)	123.6	7.35 d, (*J*= 8.1Hz)
3-O-*β*-D-glucose					3-O-*β*-D-glucose			
1′′	104.2	5.16, d (*J*= 7.2 Hz)	100.7	3.37	104.4	5.18 d, (*J*= 7.8 Hz)	104.2	4.85 d, (*J*= 7.7 Hz)
2′′	75.6	3.38 m	74.2	3.34	75.7	3.44 m	75.6	3.11-3.68 m
3′′	78.0	3.38 m	76.4	3.34	78.1	3.28 m	77.8	3.11-3.68 m
4′′	71.3	3.26 m	69.9	3.33	71.2	3.32 m	71.1	3.11-3.68 m
5′′	78.3	3.16 m	77.5	3.33	78.4	3.17 m	78.2	3.11-3.68 m
6′′	62.5	3.48, 3.63 m	60.8	3.32	62.5	3.53, 3.66 m	62.4	3.11-3.68 m

Table S1. Observed data and reported data of kaempferol-3-O-glucoside and quercetin 3-O-glucoside from *Triadica sebifera*

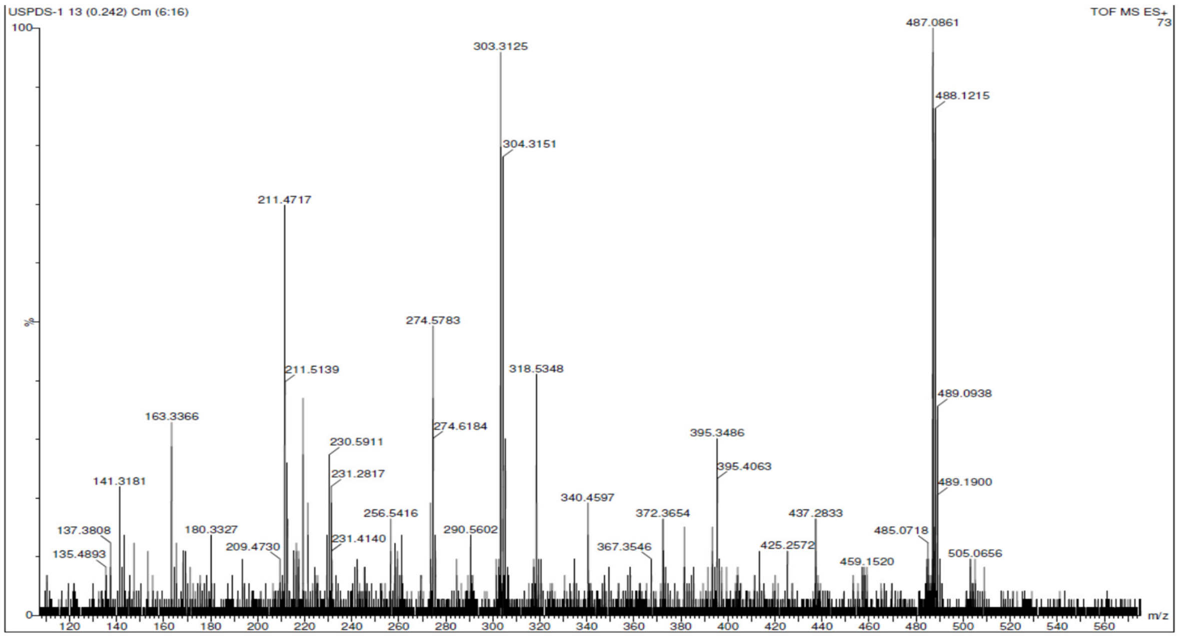

Fig. S5. ESI-MS of quercetin-3-O-glucoside

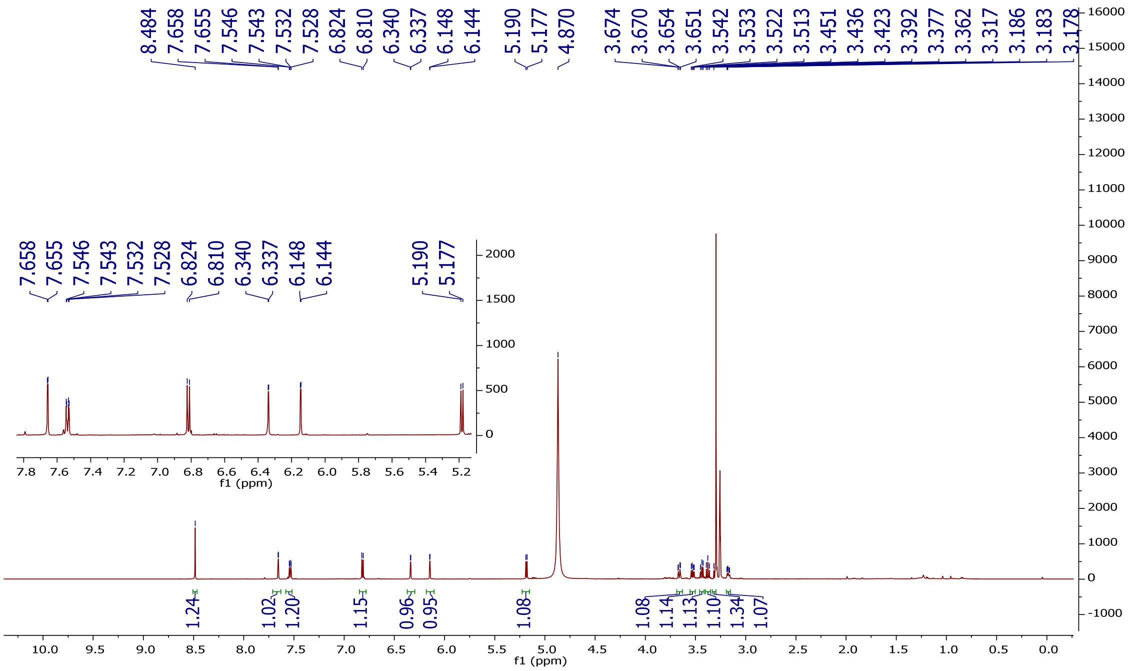

Fig. S6. ^{1}H NMR spectrum of quercetin-3-O-glucoside

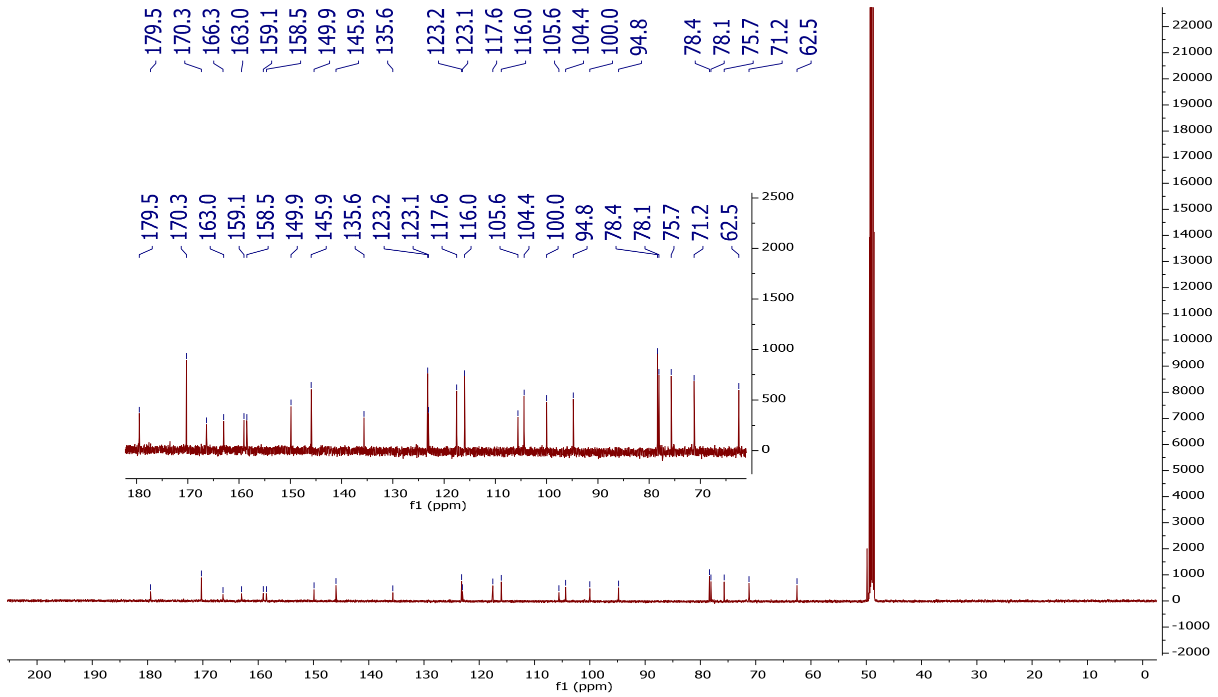

Fig. S7. ^{13}C NMR spectrum of quercetin-3-O-glucoside

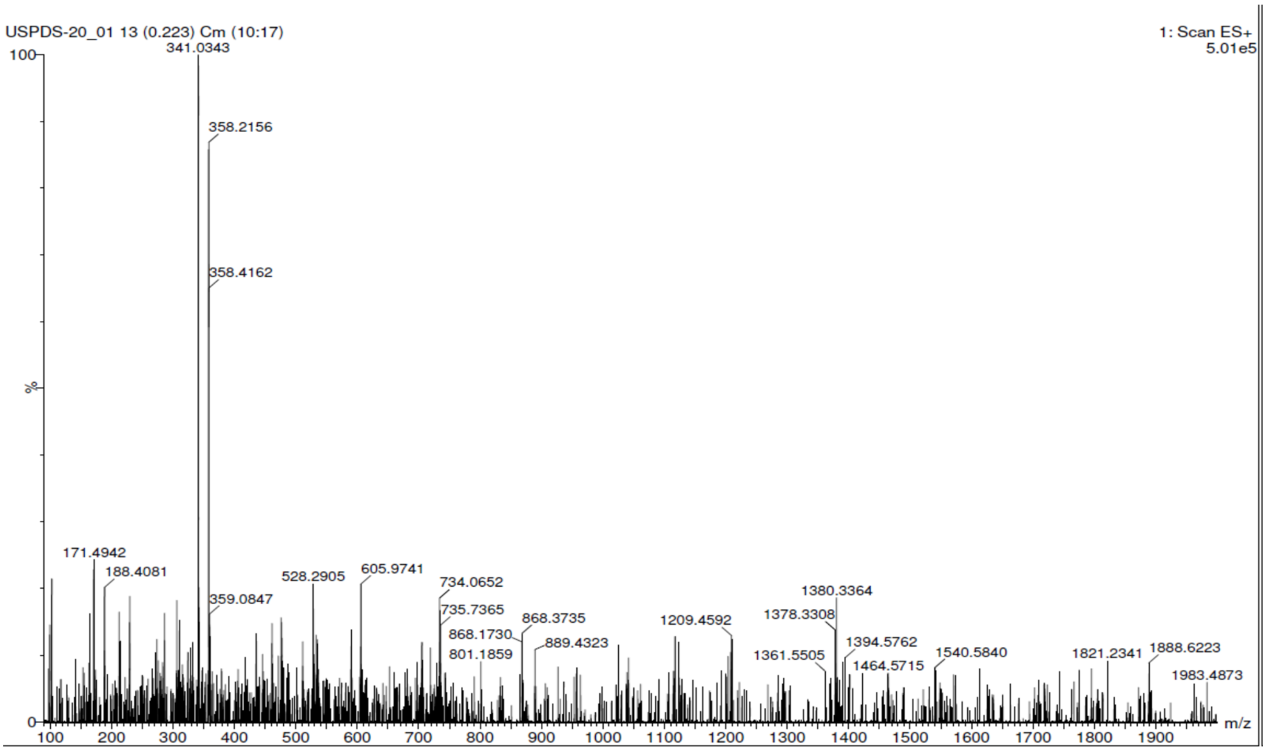

Fig. S8. ESI-MS of gallic acid

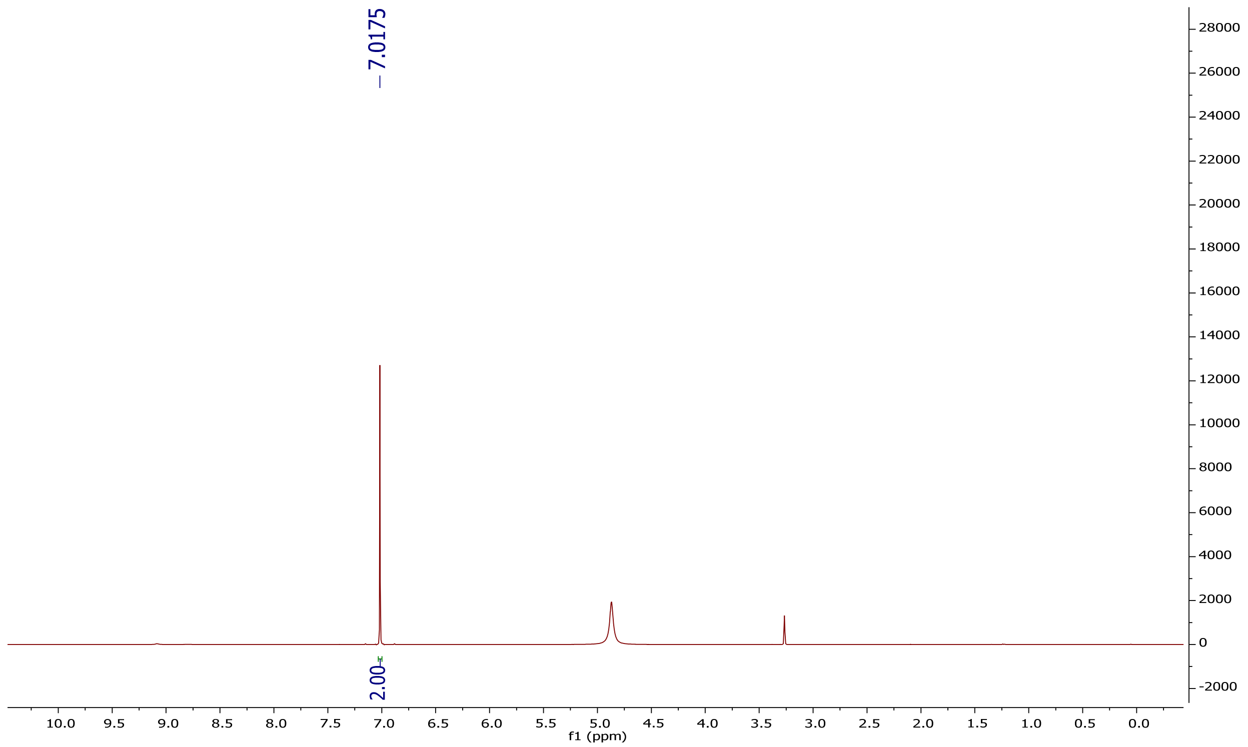

Fig. S9. ^{1}H NMR spectrum of gallic acid

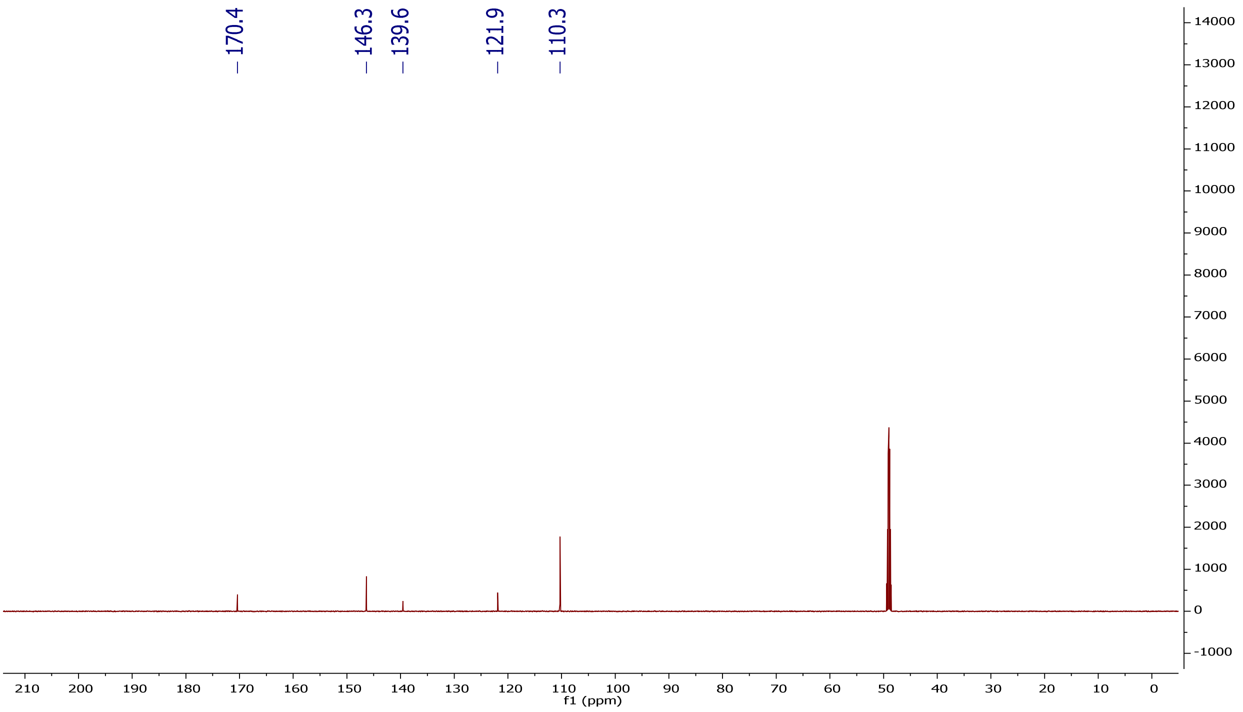

Fig. S10. ^{13}C NMR spectrum of gallic acid

Gallic acid					Shikimic acid			
Observed data			Reported data		Observed data		Reported data	
Position	δC	δH	δC	δH	δC	δH	δC	δH
1	170.4	-	167.3	-	170.0	-	170.1	-
2	121.9	-	120.8	-	130.7	-	129.8	-
3	110.3	7.01 s	109.1	7.15 s, (1H)	138.8	6.73 m, (1H)	137.1	6.70 m, (1H)
4	146.3	-	144.9	-	67.3	4.30 m, (1H)	65.8	4.30 (m, 1H)
5	139.6	-	137.7	-	72.7	3.60 (dd, *J* = 7.4, 4.2 Hz, 1H),	75.1	3.67 (dd, *J* = 8.4, 4.5 Hz, 1H)
6	146.3	-	144.9	-	68.4	3.91 m, (1H)	66.5	3.93 (m, 1H)
7	110.3	7.01 s	109.1	7.15 s, (1H)	31.6	2.12 (dd, *J* = 18.2, 5.6 Hz,1H), 2.61 (dd, *J* = 18.2, 5.0Hz, 1H),	30.4	2.12 (dd, J = 18.0, 6.3 Hz,1H), 2.64 (dd, *J* = 18.0, 4.8 Hz, 1H),

Table S2. Observed data and reported data of gallic acid and shikimic acid from *Triadica sebifera*

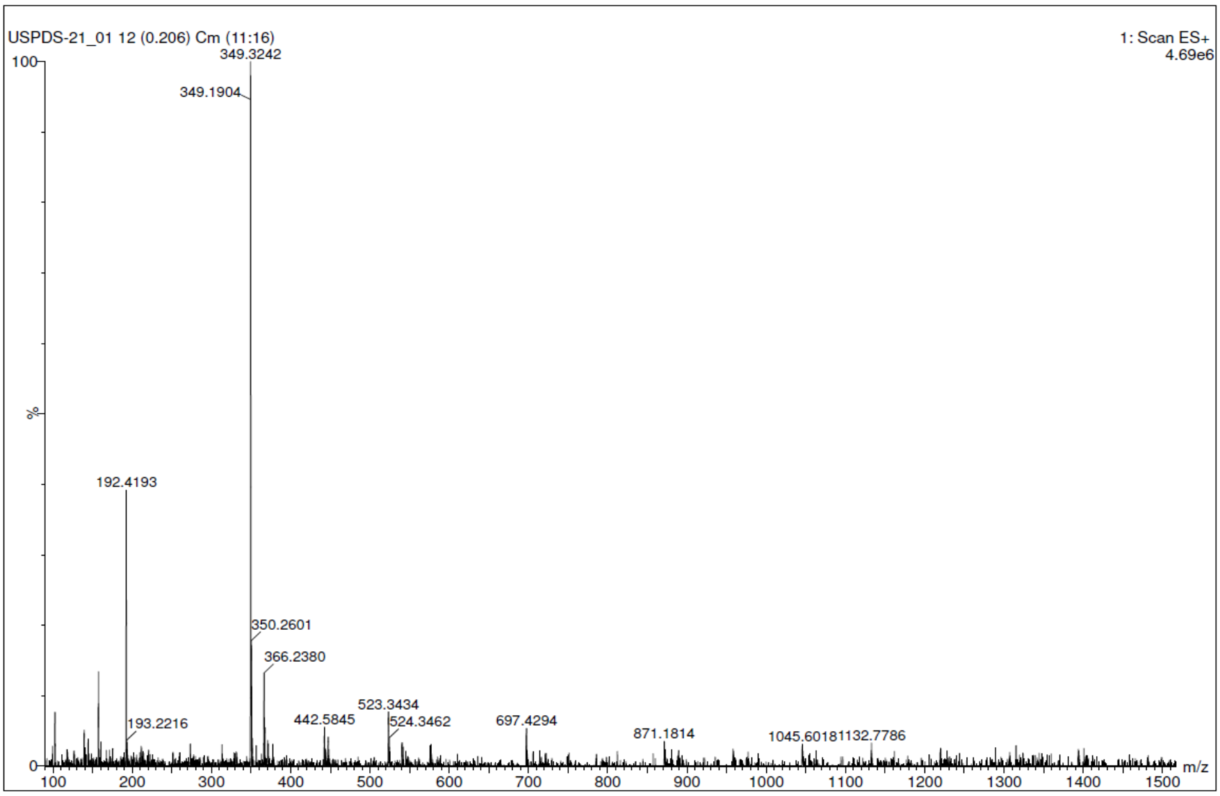

Fig. S11. ESI-MS of shikimic acid

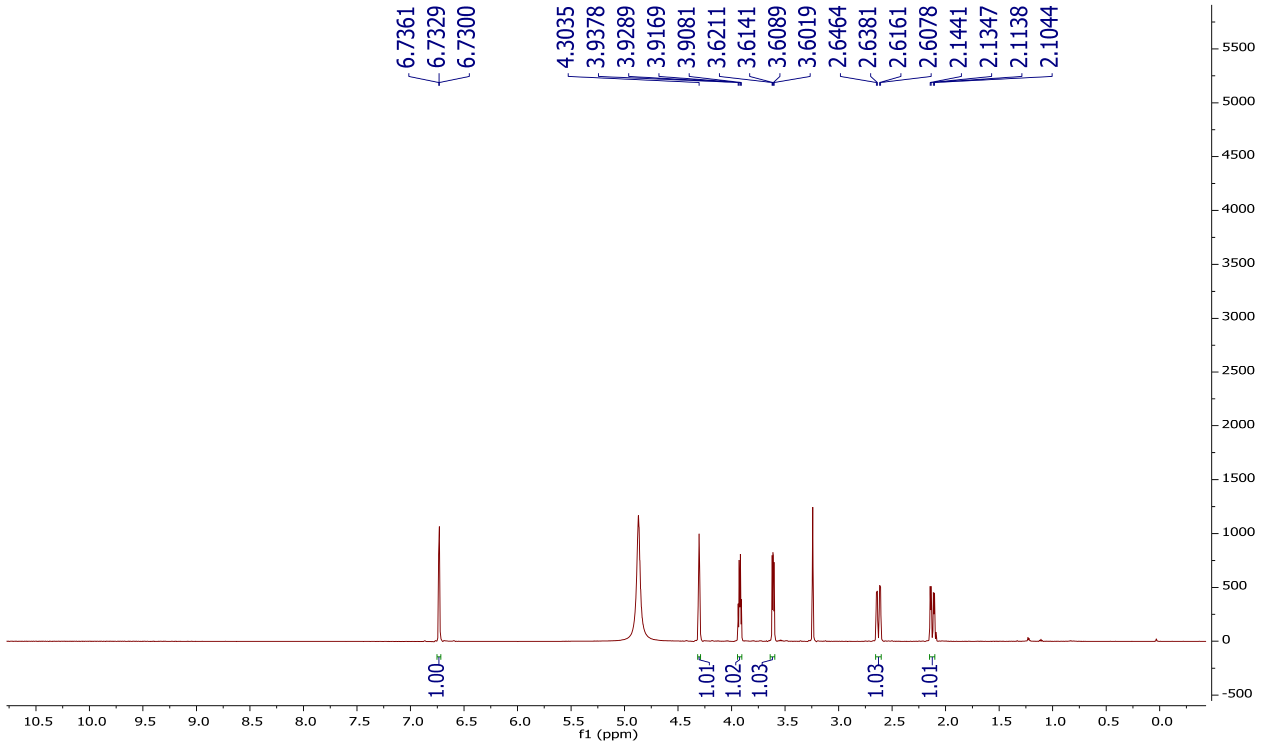

Fig. S12. ^{1}H NMR spectrum of shikimic acid

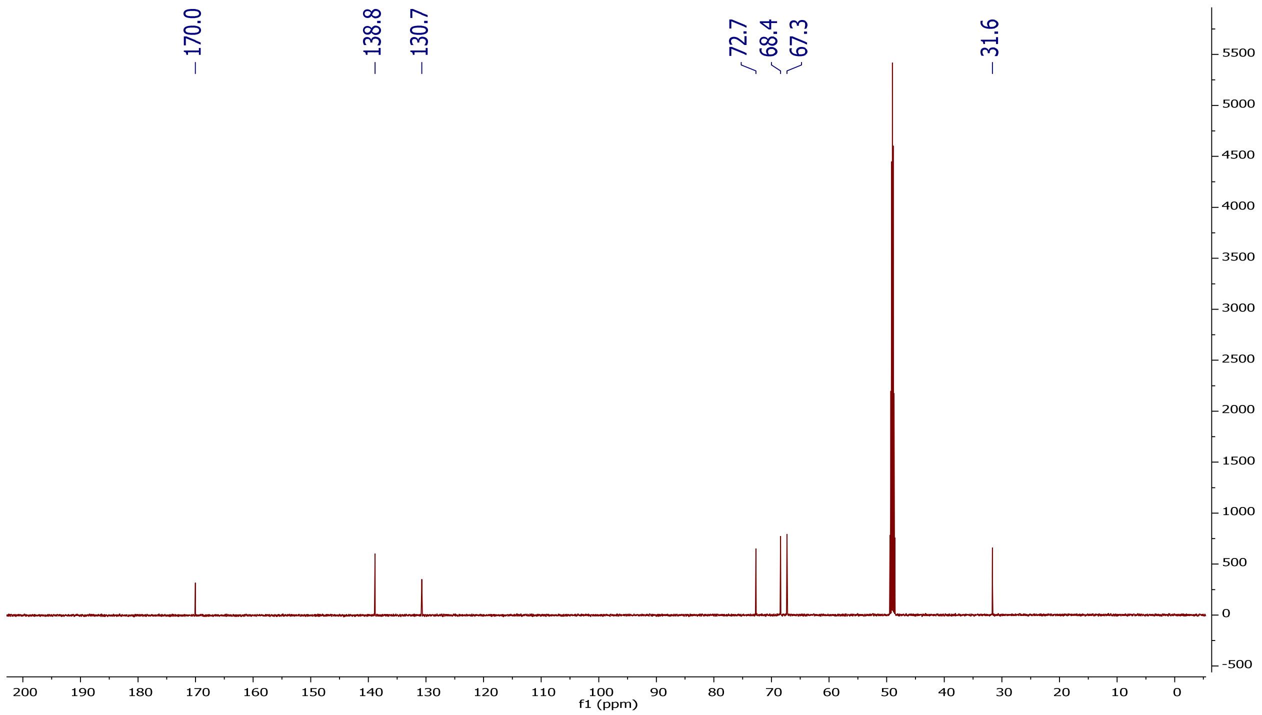

Fig. S13. ^{13}C NMR spectrum of shikimic acid

Extracts/Oil	LC_{50} * (mg/L)	Confidence limits (mg/L)	Slope ± SE	Chi square	*p* value	FEI	Interaction type
SO	2504.59	1675.92 – 3562.91	1.18 ± 0.21	0.86	0.97	–	–
LEE	9590.49	7706.54–11128.43	2.91 ± 0.52	1.81	0.87	–	–
BEE	7300.57	5889.44 – 8435.86	3.97 ± 0.57	6.17	0.29	–	–
SO: LEE (1:1)	303.29	232.07 – 403.59	1.99 ± 0.30	1.65	0.65	0.1527	Synergistic
SO: LEE (1:3)	583.34	431.14 – 901.14	1.86 ± 0.32	2.06	0.56	0.2937	Synergistic
SO: LEE (3:1)	615.35	442.80 – 1019.69	1.70 ± 0.31	0.2	0.98	0.3099	Synergistic
BEE:SO (1:1)	263.56	203.69 – 342.51	2.13 ± 0.31	0.84	0.84	0.1413	Synergistic
BEE: SO (1:3)	466.8	362.45 – 639.14	2.19 ± 0.34	0.06	0.99	0.2503	Synergistic
BEE: SO (3:1)	467.39	348.01 – 691.26	1.79 ± 0.30	0.24	0.97	0.2506	Synergistic
LEE: BEE (1:1)	508.8	393.09 – 709.19	2.17 ± 0.34	0.4	0.94	0.1227	Synergistic
LEE: BEE (1:3)	368.6	279.48 – 510.34	1.89 ± 0.30	2.08	0.56	0.0889	Synergistic
LEE: BEE (3:1)	384.95	278.66 – 579.01	1.56 ± 0.28	1.36	0.71	0.0929	Synergistic
LEE: BEE: SO (1:1:1)	337.33	259.83 – 450.11	2.04 ± 0.31	1.45	0.69	0.2161	Synergistic
(LEE+ SO): BEE (1+1: 2)	239.94	190.06 – 301.55	2.49 ± 0.34	1.4	0.71	0.1537	Synergistic
(LEE+ BEE): SO (1+1: 2)	358.53	285.84 – 461.34	2.48 ± 0.35	4.1	0.25	0.2296	Synergistic
(SO+ BEE): LEE (1+1: 2)	560.04	412.45 – 870.53	1.80 ± 0.31	0.91	0.82	0.3587	Synergistic

*LC_{50}=Lethal concentration to kill 50% of test insect; Mean of three replications; n=150 insects per treatment; LC_{50} was calculated for fractions showing > 50% mortality using Probit analysis; FEI–Fractional effect indices

Table S3. Synergistic activity of leaf/bark extracts and seed oil of *Triadica sebifera* against *Aphis craccivora* (72 h)

Extracts/oil	LC_{50} * (mg/L)	Confidence limits (mg/L)	Slope ± SE	Chi square	*p* value	FEI	Interaction type
SO	850.94	533.52 – 1171.05	1.69 ± 0.25	3.28	0.66	–	–
LEE	6756.42	5342.84 –7885.95	3.97 ± 0.58	1.84	0.87	–	–
BEE	5115.98	3613.44 – 6219.77	4.04 ± 0.75	1.31	0.73	–	–
SO: LEE (1:1)	168.9	131.54 – 211.57	2.51 ± 0.36	3.35	0.34	0.2235	Synergistic
SO: LEE (1:3)	345.05	270.79 – 451.97	2.25 ± 0.32	1.14	0.77	0.4566	Synergistic
SO: LEE (3:1)	333.88	267.82 – 422.84	2.60 ± 0.36	0.05	0.99	0.4418	Synergistic
BEE:SO (1:1)	144.26	117.51 – 174.07	3.44 ± 0.49	2.34	0.5	0.1977	Synergistic
BEE: SO (1:3)	282.49	233.42 – 344.15	3.30 ± 0.44	2.4	0.49	0.3872	Synergistic
BEE: SO (3:1)	213.8	171.19 – 265.94	2.68 ± 0.36	1.88	0.6	0.2930	Synergistic
LEE: BEE (1:1)	293.26	238.81 – 362.61	2.92 ± 0.39	0.9	0.82	0.1007	Synergistic
LEE: BEE (1:3)	179.31	146.94 – 217.38	3.28 ± 0.45	0.68	0.88	0.0616	Synergistic
LEE: BEE (3:1)	204.28	159.19 – 258.70	2.35 ± 0.33	1.12	0.77	0.0702	Synergistic
LEE: BEE: SO (1:1:1)	216.86	177.44 – 264.17	3.14 ± 0.42	1.37	0.71	0.3293	Synergistic
(LEE+ SO): BEE (1+1: 2)	170.46	137.05 – 209.14	2.94 ± 0.41	0.57	0.9	0.2589	Synergistic
(LEE+ BEE): SO (1+1: 2)	249.47	204.09 – 305.04	3.08 ± 0.41	3.21	0.36	0.3789	Synergistic
(SO+ BEE): LEE (1+1: 2)	220.58	175.94 – 275.13	2.61 ± 0.36	3.34	0.34	0.3350	Synergistic

*LC_{50}=Lethal concentration to kill 50% of test insect; Mean of three replications; n=150 insects per treatment; LC_{50} was calculated for fractions showing > 50% mortality using Probit analysis; FEI– Fractional effect indices

Table S4. Synergistic activity of leaf/bark extracts and seed oil of *Triadica sebifera* against *Aphis craccivora* (96 h)

Oil/ Extracts	LC_{50} * (mg/L)	Confidence limits (mg/L)	Slope ± SE	Chi square	*p* value	FEI	Interaction type
SO (72 h)	1100.22	812.86 – 1392.09	1.85 ± 0.23	2.88	0.58	–	–
SO (96 h)	706.53	532.34 – 976.85	2.06 ± 0.28	1.02	0.91	–	–
LEE (72 h)	1840.84	1199.35 – 2565.63	1.07 ± 0.16	4.44	0.35	–	–
LEE (96 h)	685.47	442.65 – 917.21	1.82 ± 0.26	1.45	0.83	–	–
BEE (72 h)	5073.99	3982.57 – 6609.45	1.50 ± 0.17	4.45	0.35	–	–
BEE (96 h)	2328.79	1966.60 –2741.25	2.68 ± 0.26	2.79	0.59	–	–
SO + LEE (72 h)	264.05	210.93 – 319.75	2.27 ± 0.27	3.03	0.39	0.383	Synergistic
SO + LEE (96 h)	223.82	178.50 – 269.53	2.49 ± 0.31	2.44	0.49	0.643	Additive
SO + BEE (72 h)	362.76	293.43 – 440.43	2.14 ± 0.25	1.94	0.58	0.401	Synergistic
SO + BEE (96 h)	247.54	201.52 – 295.38	2.58 ± 0.31	1.33	0.72	0.456	Synergistic

* LC_{50}=Lethal concentration to kill 50% of test insect; Mean of three replications; n=150 insects per treatment; LC_{50} was calculated for fractions showing > 50% mortality using Probit analysis; FEI–Fractional effect indices

Table S5. Efficacy of leaf/bark ethanolic aqueous extracts and seed oil of *Triadica sebifera* against *Aphis craccivora* under plant growth chamber

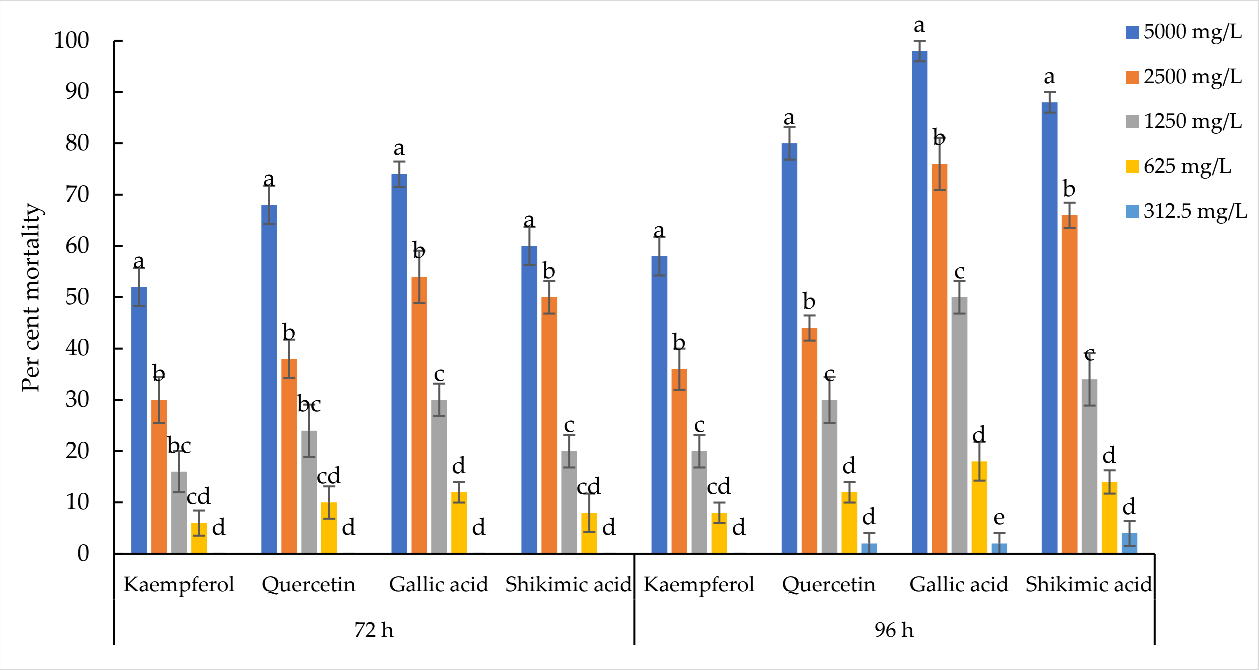

Fig. S14. Bio-efficacy of isolated compounds from leaf fractions of *Triadica sebifera* against *Aphis craccivora*; Figures in the same alphabetical letters in the error bars are statistically at par by Tukey's HSD ($p \geq 0.05$)
